# A comprehensive review on magnetic manganese as catalysts in organic synthesis 

**DOI:** 10.1039/d5ra02493e

**Published:** 2025-07-07

**Authors:** Mosstafa Kazemi, Radwan Ali, Vicky Jain, Suhas Ballal, Munthar Kadhim Abosaoda, Abhayveer Singh, T. Krithiga, Kamal Kant Joshi, Ramin Javahershenas

**Affiliations:** a Young Researchers and Elite Club, Tehran Branch, Islamic Azad University Tehran Iran mosstafakazemi@gmail.com; b Al-Qadisiyah University, College of Dentistry, Department of Basic Sciences Al-Qadisiyah Iraq radwan.ali@qu.edu.iq; c Marwadi University Research Center, Department of Chemistry, Faculty of Science Marwadi University Rajkot-360003 Gujarat India vicky.jain@marwadieducation.edu.in; d Department of Chemistry and Biochemistry, School of Sciences, JAIN (Deemed to be University) Bangalore Karnataka India b.suhas@jainuniversity.ac.in; e College of Pharmacy, The Islamic University Najaf Iraq; f College of Pharmacy, The Islamic University of Al Diwaniyah Al Diwaniyah Iraq muntherabosoda@iunajaf.edu.iq; g Centre for Research Impact & Outcome, Chitkara University Institute of Engineering and Technology, Chitkara University Rajpura 140401 Punjab India abhayveer_singh@outlook.com; h Department of Chemistry, Sathyabama Institute of Science and Technology Chennai Tamil Nadu India krithiga.chemistry@sathyabama.ac.in; i Department of Allied Science, Graphic Era Hill University Dehradun India; j Graphic Era Deemed to be University Dehradun Uttarakhand India kkjoshi@gehu.ac.in; k Young Researchers and Elite Club, Tehran Branch, Islamic Azad University Tehran Iran jshbco@yahoo.com

## Abstract

Manganese-based magnetic catalysts have gained significant attention in modern catalysis due to their unique combination of high catalytic efficiency, magnetic recoverability, and environmental sustainability. These catalysts, typically composed of manganese oxides, manganese-doped ferrites, or Mn-functionalized magnetic nanoparticles, facilitate a wide range of chemical transformations, including oxidation reactions, coupling reactions, and multicomponent reactions especially in the synthesis of heterocycles. Their ability to exhibit multiple oxidation states, strong redox activity, and high surface area makes them highly effective in selective and energy-efficient catalytic processes. Additionally, their magnetic properties enable easy separation from reaction mixtures using an external magnetic field, improving catalyst recyclability and reducing operational costs. Compared to conventional catalysts, magnetic manganese catalysts offer superior stability, cost-effectiveness, and eco-friendliness, making them promising alternatives for industrial-scale applications. This review explores recent advancements in the synthesis, mechanistic insights, and diverse applications of magnetic manganese catalysts, highlighting their role in sustainable and green chemistry. Furthermore, the challenges and future perspectives in optimizing their performance for broader catalytic applications are discussed. The insights presented in this review underscore the growing importance of magnetic manganese catalysts in developing efficient, cost-effective, and environmentally benign catalytic systems.

## Abstract 2

Manganese-based magnetic catalysts have attracted considerable interest due to their high catalytic efficiency, magnetic recyclability, and eco-friendly nature in the synthesis of heterocycles.

## Graphical abstract

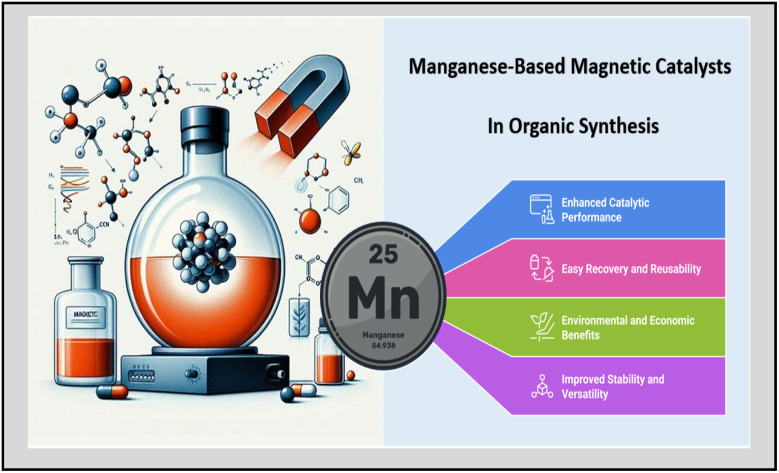

## 1 Introduction

Catalysts play a crucial role in chemistry, particularly in the synthesis of organic compounds, by accelerating chemical reactions without being consumed in the process.^1^ They lower the activation energy required for reactions to occur, increasing efficiency and selectivity while reducing energy consumption and waste.^2,3^ In industrial and pharmaceutical applications, catalysts enable the large-scale production of essential chemicals, fuels, and medicines with higher yields and fewer byproducts.^4^ Enzymes, metal complexes, and solid-state catalysts are widely used to facilitate complex transformations, such as hydrogenation, polymerization, and oxidation.^5–7^ Their significance extends to green chemistry, where they help develop sustainable and environmentally friendly synthetic processes.^8^

### 1.1. Homogeneous and heterogeneous catalysis

**Fig. 1:**
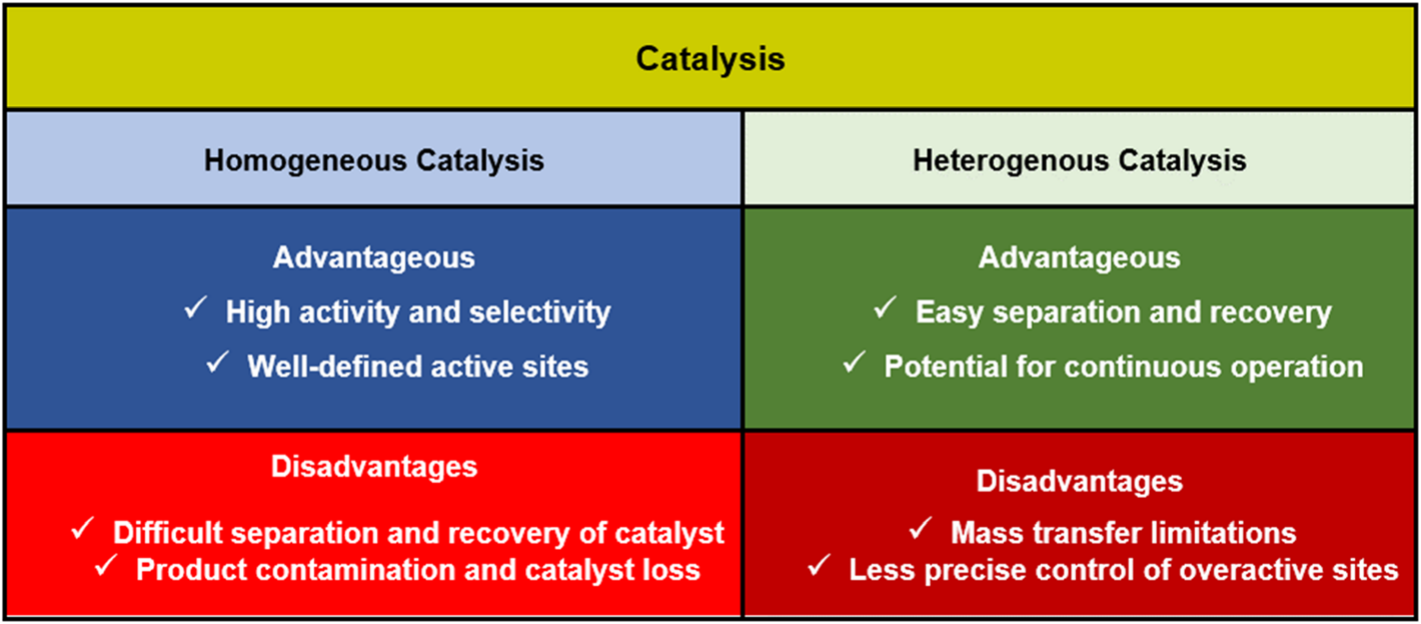
Catalysis by homogenous and heterogenous catalysts.

In chemical reactions, catalysts are categorized into two types: homogeneous and heterogeneous catalysts.^9^ Homogeneous and heterogeneous catalysts both play essential roles in chemical reactions, but they differ in efficiency based on factors like reaction conditions, selectivity, and reusability (Fig. 1).^10^

#### 1.1.1. Homogeneous catalysts

Homogeneous catalysts are in the same phase as the reactants, typically dissolved in a liquid medium. This allows for better molecular interactions, leading to higher reaction rates and selectivity.^11^ They are particularly effective in fine chemical and pharmaceutical synthesis, where precise control over reaction pathways is crucial. However, their major drawback is difficulty in separation and recovery, making them less practical for large-scale industrial applications.^12,13^

#### 1.1.2. Heterogeneous catalysts

Heterogeneous catalysts, on the other hand, exist in a different phase than the reactants, usually as solid catalysts interacting with gaseous or liquid reactants.^14^ Their biggest advantage is ease of separation and recyclability, making them ideal for large-scale industrial processes like petroleum refining and environmental catalysis.^15^ While they generally offer lower selectivity and slower reaction rates due to mass transfer limitations, their stability and ability to operate under extreme conditions make them highly efficient for bulk chemical production.^16^ Overall, homogeneous catalysts are superior in precision and reaction speed, while heterogeneous catalysts excel in practicality, sustainability, and large-scale applications.^17^

### 1.2. Catalysis by nanomaterials

Nanomaterials offer significant advantages in catalyzing chemical reactions due to their exceptionally high surface area-to-volume ratio.^18,19^ This increased surface area provides more active sites for reactant molecules to interact with, enhancing the reaction rate and efficiency.^20^ Additionally, the small size of nanoparticles allows for better dispersion in reaction media, improving accessibility to catalytic sites and reducing the amount of material needed for effective catalysis.^21^ Their unique electronic and structural properties, which differ from their bulk counterparts, can also lead to enhanced catalytic activity, selectivity, and stability in various chemical processes.^22^

Another key advantage of nanomaterials in catalysis is their tunability. By modifying their size, shape, composition, and surface chemistry, scientists can precisely control their catalytic behavior to optimize reaction pathways and minimize unwanted byproducts.^23^ For example, metal nanoparticles, such as gold and platinum, can be engineered at the nanoscale to exhibit superior catalytic performance in energy-related applications like fuel cells and hydrogen production.^24^ Moreover, nanomaterials enable the development of environmentally friendly catalytic systems by reducing energy consumption and replacing hazardous catalysts with more sustainable alternatives.^25^ These benefits make nanomaterial-based catalysts highly valuable in industrial processes, green chemistry, and emerging technologies.

### 1.3. Magnetic nanocatalysts

Magnetic nanocatalysts represent an innovative approach in catalysis, combining the benefits of both homogeneous and heterogeneous systems while offering unique advantages in reaction efficiency and catalyst recovery.^26–28^ These catalysts typically consist of magnetic nanoparticles, often made from iron-based materials such as magnetite (Fe_3_O_4_) or other transition metal oxides, functionalized with catalytic sites.^29,30^ The field of magnetic catalysis has emerged from the need for sustainable and easily recoverable catalytic systems, particularly in industries such as pharmaceuticals, petrochemicals, and green chemistry.^31,32^

The development of magnetic catalysts was driven by the limitations of traditional homogeneous and heterogeneous catalysis.^33,34^ Homogeneous catalysts, while highly selective and efficient, are difficult to separate from the reaction mixture, leading to high costs and environmental concerns.^35^ Heterogeneous catalysts, on the other hand, are easier to recover but often suffer from lower reaction rates due to limited surface area and mass transfer constraints.^36,37^ Magnetic catalysts bridge this gap by providing high catalytic activity while allowing for easy separation using an external magnetic field, eliminating the need for filtration or centrifugation.^38,39^

#### 1.3.1. Advantages of magnetic catalysts

Easy recovery and reusability: the most significant advantage of magnetic catalysts is their ability to be separated from reaction mixtures simply by applying an external magnetic field.^40^ This eliminates energy-intensive separation processes such as filtration, centrifugation, or solvent extraction, making them highly cost-effective and environmentally friendly.^38,41^

High surface area and enhanced catalytic activity: magnetic nanoparticles provide a large surface area for catalytic reactions, which increases reaction rates and efficiency.^42,43^ Functionalization with active catalytic sites, such as metal complexes or enzymes, further enhances their activity.^44^

Improved stability and durability: many magnetic catalysts are designed to be chemically and thermally stable, allowing them to withstand harsh reaction conditions.^45^ This extends their lifespan, reducing the need for frequent catalyst replacement.^46^

Versatility in chemical transformations: magnetic catalysts have been successfully applied in various chemical reactions, including oxidation, hydrogenation, coupling reactions, and organic transformations.^47^ Their adaptability to different reaction conditions makes them highly valuable in diverse industrial and laboratory settings.^48,49^

Environmental and economic benefits: by reducing waste, minimizing the use of toxic solvents, and lowering energy consumption, magnetic catalysts contribute to greener and more sustainable chemical processes.^50^ Their reusability also reduces costs, making them an attractive alternative for industrial applications.^51^

**Fig. 2:**
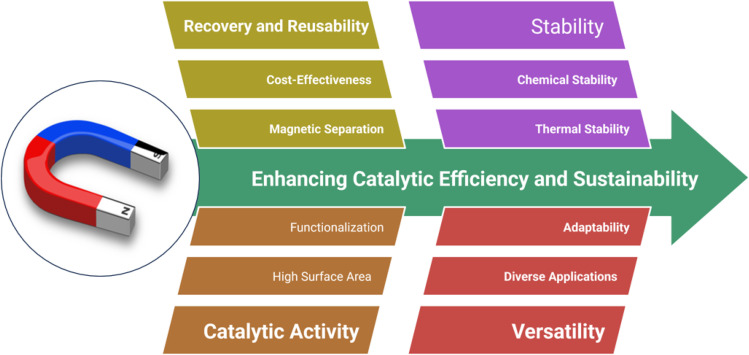
Advantages of magnetic nanocatalysts.

Overall, magnetic catalysts are a promising advancement in catalysis, offering an efficient, cost-effective, and sustainable approach to carrying out chemical reactions while addressing some of the key limitations of traditional catalytic systems (Fig. 2).^52^

#### 1.3.2. Comparison between magnetic nanocatalysts and conventional catalysts

Magnetic nanocatalysts offer several advantages over conventional catalysts, particularly in terms of efficiency, recovery, and sustainability. The following comparison highlights key differences between these catalytic systems (Table 1).

**Table 1:** Comparison between magnetic nanocatalysts and conventional catalysts

Feature	Magnetic catalysts	Conventional catalysts
Efficiency	High efficiency due to large surface area and ease of functionalization. Can be tailored for specific reactions	Efficiency varies; homogeneous catalysts offer high selectivity but are hard to recover, while heterogeneous catalysts may have lower surface interaction
Recovery & reusability	Easily recovered using an external magnetic field, reducing waste and energy costs. Highly reusable	Homogeneous catalysts are difficult to separate and often lost, while heterogeneous catalysts require filtration or centrifugation for recovery
Catalyst stability	More stable under harsh conditions, reducing catalyst deactivation over time	Stability depends on the catalyst type; homogeneous catalysts degrade faster, while some heterogeneous catalysts are more robust
Reaction rates	Comparable or higher than conventional catalysts due to increased surface area and optimized functionalization	Homogeneous catalysts offer faster reaction rates, but heterogeneous catalysts can suffer from diffusion limitations
Selectivity	Can be designed for high selectivity using functionalized nanoparticles and controlled reaction conditions	Homogeneous catalysts provide excellent selectivity, while heterogeneous catalysts may require optimization for specificity
Environmental impact	More sustainable due to easy recovery, reduced waste, and lower energy consumption. Supports green chemistry initiatives	Conventional catalysts often require toxic solvents, excess reagents, and complex separation steps, increasing environmental impact
Industrial application	Highly suitable for scalable industrial applications due to reusability and low separation costs	Used in various industries, but recovery challenges and process inefficiencies can increase costs
Cost-effectiveness	Lower long-term costs due to reusability and easy separation, though initial synthesis may be more expensive	Homogeneous catalysts can be expensive due to loss during reactions, while heterogeneous catalysts require periodic regeneration or replacement
Catalyst stability	More stable under harsh conditions, reducing catalyst deactivation over time	Stability depends on the catalyst type; homogeneous catalysts degrade faster, while some heterogeneous catalysts are more robust
Reaction rates	Comparable or higher than conventional catalysts due to increased surface area and optimized functionalization	Homogeneous catalysts offer faster reaction rates, but heterogeneous catalysts can suffer from diffusion limitations

#### 1.3.3. Overall efficiency and advantages

✓ Magnetic nanocatalysts provide a balance between homogeneous and heterogeneous catalysts, offering high efficiency, easy recovery, and environmental benefits.^53^

✓ Magnetic separation: conventional catalysts (especially homogeneous ones) can sometimes have higher selectivity but suffer from difficult separation and loss, leading to increased costs and waste.^54^

✓ Magnetic nanocatalysts are particularly advantageous for sustainable and large-scale industrial applications, as they reduce energy consumption and environmental impact while maintaining high catalytic performance.^55^

In summary, while conventional catalysts remain widely used, magnetic catalysts offer a superior alternative in many cases due to their recyclability, efficiency, and eco-friendly nature.

### 1.4. Catalysis by transition metals

Transition metals play a vital role as catalysts in chemical reactions due to their unique electronic structures and ability to adopt multiple oxidation states.^56^ Their partially filled d-orbitals allow them to form transient complexes with reactants, stabilizing intermediate species and lowering activation energy.^57^ This property enhances reaction rates and selectivity, making transition metal catalysts indispensable in both industrial and laboratory-scale chemistry.^58^ Common transition metals like platinum, palladium, iron, and copper are widely used in catalytic processes such as hydrogenation, oxidation, and carbon–carbon bond formation, significantly improving reaction efficiency while minimizing energy consumption and waste production.^59^

In industrial applications, transition metal catalysts are essential for the large-scale production of chemicals, fuels, and pharmaceuticals.^60^ For instance, platinum and rhodium are used in catalytic converters to reduce harmful vehicle emissions, while nickel and cobalt facilitate hydrogenation reactions in the food and petrochemical industries.^61^ Additionally, transition metals are fundamental in green chemistry, as they enable more sustainable processes by reducing the need for harsh reaction conditions and toxic reagents.^58^ Their role in organometallic catalysis, particularly in cross-coupling reactions like the Suzuki and Heck reactions, has revolutionized the synthesis of complex organic molecules, including pharmaceuticals and advanced materials.^62^ Thus, transition metal catalysts continue to drive innovation in chemical research and industrial production.

### 1.5. Catalysis by manganese

Manganese and its compounds have emerged as highly efficient catalysts for a wide range of chemical reactions due to their diverse oxidation states, affordability, and environmental sustainability.^63^ As a first-row transition metal, manganese exhibits oxidation states ranging from +2 to +7, making it highly versatile in catalytic processes such as oxidation, reduction, and organometallic transformations.^64^ Its ability to form stable yet reactive complexes allow for controlled and selective chemical reactions, making it a valuable catalyst in industrial and laboratory settings.

#### 1.5.1. Applications of manganese-based catalysts

❖ Oxidation reactions

✓ Manganese-based catalysts are widely used in oxidation reactions, particularly in the oxidation of alcohols, hydrocarbons, and organic substrates.^65^

✓ Manganese dioxide (MnO_2_) is an efficient catalyst for oxidation reactions, commonly used in organic synthesis for oxidizing primary and secondary alcohols to aldehydes and ketones.^66^

✓ Manganese porphyrins and salen complexes mimic the function of metalloenzymes and serve as highly selective catalysts in oxidation reactions, including epoxidation and hydroxylation.^67^

❖ Water oxidation and electrocatalysis

✓ Manganese oxides play a crucial role in artificial photosynthesis and water-splitting reactions, aiding in the oxidation of water to oxygen.^68^

✓ Mn-based catalysts are extensively studied in electrocatalysis for energy conversion applications, such as fuel cells and metal–air batteries. Their low-cost and earth-abundant nature make them ideal substitutes for noble metal catalysts like platinum and ruthenium.^69^

❖ C–C and C–X bond formation

✓ Manganese-catalyzed cross-coupling reactions enable efficient carbon–carbon and carbon–heteroatom bond formation, essential in pharmaceutical and fine chemical synthesis.^70^

✓ Mn(iii) and Mn(v) complexes are known to facilitate various radical-mediated transformations, making them valuable tools in modern synthetic organic chemistry.^71^

❖ Green and sustainable catalysis

✓ Manganese catalysts contribute significantly to sustainable chemistry by promoting reactions under mild conditions and minimizing the use of toxic reagents.^72^

✓ Mn-based catalysts are utilized in bioinspired oxidation processes and asymmetric synthesis, reducing the environmental impact compared to traditional catalytic systems.^73^

❖ Industrial and environmental applications

✓ Mn-based composites are used in catalytic converters to decompose harmful pollutants and reduce emissions from industrial processes.^74^

✓ Manganese-catalyzed oxidation of pollutants in wastewater treatment and air purification highlights its environmental significance.^75^

#### 1.5.2. Superiority of manganese as a catalyst over other transition metals

**Fig. 3:**
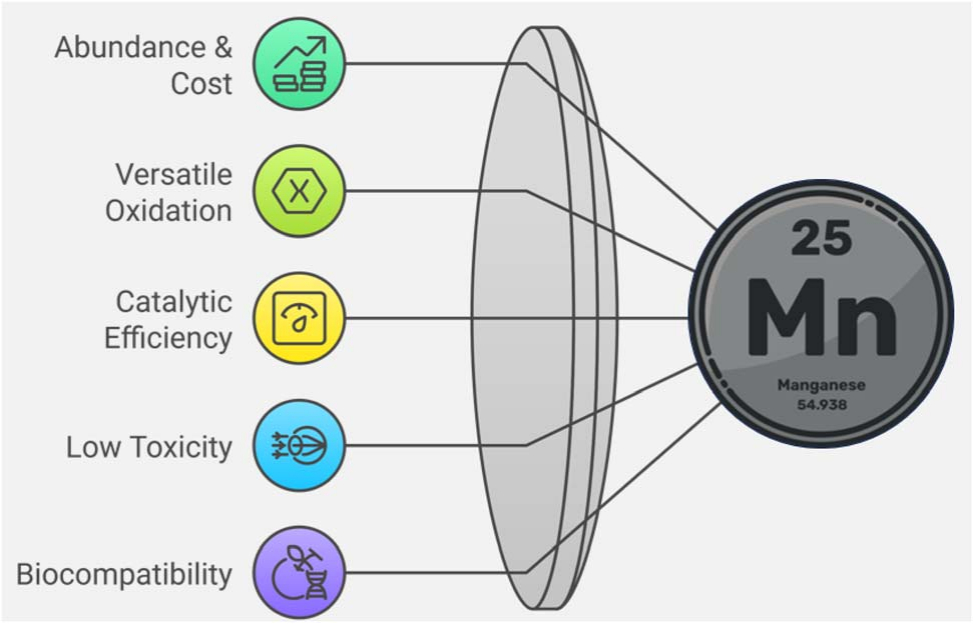
Superiority of manganese over other transition metals in catalysis.

Manganese holds several advantages over other transition metals in catalysis, making it a superior choice for numerous chemical transformations (Fig. 3).

❖Abundance and cost-effectiveness

✓ Manganese is the 12th most abundant element on Earth and is significantly more affordable than precious metals like platinum, palladium, rhodium, and ruthenium.^76^

✓ The low cost of manganese compounds allows for their widespread use in both academic research and industrial applications.^77^

❖ Versatile oxidation states and redox activity

✓ Unlike many transition metals that primarily operate in a limited number of oxidation states, manganese exhibits oxidation states from +2 to +7, enabling a broad range of catalytic functions.^78^

✓ This redox flexibility makes manganese an excellent catalyst for oxidation and reduction reactions, including radical-mediated processes that are difficult to achieve with other metals.^79^

❖ High catalytic efficiency and selectivity

✓ Manganese-based catalysts often achieve high turnover numbers (TON) and turnover frequencies (TOF), leading to efficient catalytic cycles.^80^

✓ Mn(iii) and Mn(v)-oxo species exhibit superior selectivity in oxidation reactions compared to iron and copper catalysts, reducing the formation of undesired byproducts.^81^

❖ Green chemistry and low toxicity

✓ Unlike heavy metals such as mercury, lead, and cadmium, manganese compounds are generally non-toxic and environmentally benign.^82^

✓ The use of manganese catalysts aligns with the principles of green chemistry, reducing hazardous waste and allowing for sustainable reaction pathways.^83^

❖ Biocompatibility and biomimetic applications

✓ Manganese is an essential element in biological systems, found in metalloenzymes such as Mn-superoxide dismutase and oxygen–evolving complex (OEC) in photosystem II.^84^

✓ Manganese-based biomimetic catalysts are extensively used to replicate enzymatic transformations, offering eco-friendly solutions for pharmaceutical and biochemical applications.^85^

As a result, manganese and its compounds are indispensable in modern catalysis due to their redox versatility, cost-effectiveness, high efficiency, and environmental sustainability. Their ability to facilitate key transformations, from oxidation and electrocatalysis to cross-coupling and green chemistry, makes them a superior alternative to expensive noble metal catalysts.^86^ Additionally, manganese's abundance, low toxicity, and biomimetic properties reinforce its significance as a catalyst in both fundamental and applied chemistry. As research in catalysis advances, manganese-based catalytic systems will continue to play a crucial role in developing efficient, sustainable, and economically viable chemical processes.

## 2 Catalysis by manganese magnetic nanocatalysts

Magnetic catalysts represent a significant advancement in catalysis, offering high efficiency, easy separation, and recyclability. Manganese-based magnetic catalysts combine the advantages of manganese catalysis and magnetic nanomaterials, making them an attractive choice for sustainable and industrial-scale applications.^87^ These catalysts are particularly useful in oxidation reactions, organic transformations, electrocatalysis, and environmental remediation. Their magnetic properties allow for simple and efficient recovery using an external magnetic field, reducing waste and improving cost-effectiveness.^88^

Highlights of manganese magnetic nanocatalysts

❖ Enhanced catalytic performance

✓ Magnetic manganese catalysts exhibit high surface area, improving catalytic activity.

✓ The synergistic effect between Mn and magnetic components (*e.g.*, Fe_3_O_4_) enhances redox reactions and selectivity.

❖ Easy recovery and reusability

✓ The magnetic nature of these catalysts allows for effortless separation using an external magnetic field, eliminating the need for filtration or centrifugation.

✓ This improves catalyst stability and recyclability, reducing material costs and waste generation.

❖ Environmental and economic benefits

✓ Manganese is abundant and inexpensive, making Mn-based catalysts a cost-effective alternative to noble metal catalysts.

✓ Mn magnetic catalysts enable green chemistry practices by reducing hazardous reagent use, lowering energy consumption, and promoting cleaner reaction processes.

❖ Improved stability and versatility

✓ Mn-based catalysts maintain their activity over multiple cycles without significant deactivation.

✓ Their ability to function under mild or extreme reaction conditions makes them suitable for diverse industrial applications.

As a result, magnetic manganese catalysts offer a highly efficient, cost-effective, and environmentally friendly alternative to conventional catalytic systems (Table 2). Their ability to catalyze oxidation reactions, organic transformations, and electrocatalytic processes-while being easily recoverable and reusable-makes them invaluable in sustainable chemistry and industrial applications.^89^ With advancements in catalyst design, Mn-based magnetic catalysts will continue to drive innovation in green catalysis, energy storage, and environmental protection.^90^

**Table 2:** Advantages of catalysis by magnetic manganese catalysts

Advantages	Explanation
High catalytic efficiency	Manganese's multiple oxidation states facilitate fast and selective reactions
Magnetic separation	Easy recovery reduces processing costs and energy consumption
Reusability	Can be used multiple times without loss of activity, enhancing sustainability
Eco-friendly	Reduces toxic reagent use and supports green chemistry
Cost-effectiveness	Manganese is cheaper and more abundant than noble metals like palladium or platinum
Industrial scalability	Suitable for large-scale applications due to stability and easy recovery

Magnetic manganese catalysts have emerged as versatile tools in both drug synthesis and environmental remediation, offering unique advantages in efficiency, recyclability, and sustainability. In pharmaceutical applications, these catalysts enable critical transformations while simplifying workflows. For instance, the oxidation of secondary alcohols to ketones—a pivotal step in synthesizing intermediates for drugs like tamoxifen and ibuprofen—can be efficiently mediated by MnO_2_ supported on magnetic Fe_3_O_4_@SiO_2_ nanoparticles. Unlike traditional stoichiometric oxidants, this system avoids excessive waste generation and allows rapid catalyst recovery *via* external magnets, making it reusable for over 10 cycles without significant activity loss. Similarly, in asymmetric epoxidation reactions, manganese–porphyrin complexes anchored to magnetic carbon-coated nanoparticles mimic enzymatic precision, generating chiral epoxides essential for β-blockers such as propranolol. The magnetic support not only stabilizes the catalyst but also circumvents labor-intensive purification steps like chromatography, streamlining the synthesis of enantiopure pharmaceuticals. Further expanding their utility, magnetic manganese oxides facilitate direct C–H amination of aromatic compounds, a reaction critical to antiviral agents like oseltamivir (Tamiflu). By replacing precious metal catalysts, these systems reduce costs and eliminate risks of heavy metal contamination in final drug products.

In environmental applications, magnetic manganese catalysts address pressing challenges in industrial wastewater treatment. For example, MnFe_2_O_4_ nanoparticles degrade persistent azo dyes from textile effluents through Fenton-like reactions, generating hydroxyl radicals that break down pollutants at neutral pH—a significant improvement over classical Fenton processes requiring acidic conditions. The magnetic core ensures nearly complete catalyst retrieval after treatment, preventing secondary pollution from nanoparticle discharge. Similarly, Mn_3_O_4_/Fe_3_O_4_@SiO_2_ composites activate peroxymonosulfate to degrade antibiotics like tetracycline, achieving over 90% removal efficiency even in complex wastewater matrices. This approach minimizes sludge generation and operational downtime, as magnetic separation replaces cumbersome filtration. In petrochemical wastewater, MnO_2_/Fe_3_O_4_ hybrids mineralize toxic phenols into harmless CO_2_ and water *via* catalytic wet peroxide oxidation, operating efficiently under ambient conditions without producing hazardous byproducts.

While both drug synthesis and wastewater treatment benefit from magnetic design, their applications diverge in focus. In pharmaceuticals, the emphasis lies on enhancing selectivity and reducing contamination, whereas environmental applications prioritize pollutant mineralization and scalability. Challenges remain, such as mitigating manganese leaching in acidic drug synthesis conditions or preventing catalyst fouling in wastewater.^196–198^ However, innovations like graphene coatings and hydrophilic functionalizations are paving the way for robust, long-lasting systems. Despite higher initial synthesis costs, magnetic manganese catalysts prove economically viable over time due to their reusability and alignment with green chemistry principles. As industries increasingly prioritize sustainability, these catalysts are poised to play a transformative role in sustainable drug manufacturing and large-scale water remediation, bridging the gap between laboratory innovation and industrial practicality.^199–201^

Continuing our research on magnetic catalysts, this review explores the advantages of employing magnetic catalysts in chemical reactions including oxidation, coupling reactions and synthesis of heterocycles, emphasizing their enhanced catalytic activity, ease of separation, and environmental sustainability.

### 2.1. Oxidation reactions

Oxidation reactions are fundamental to organic chemistry, serving as pivotal processes that influence the reactivity, stability, and transformation of organic compounds.^91^ Oxidation typically involves the loss of electrons or an increase in oxidation state, resulting in the modification of molecular structures and the introduction of functional groups.^92^ These reactions are not only essential for synthetic applications but also play a critical role in biological systems. One of the most significant aspects of oxidation reactions in organic chemistry is their application in synthetic pathways. Various oxidation techniques enable chemists to convert readily available starting materials into more complex molecules, facilitating the synthesis of pharmaceuticals, agrochemicals, and natural products.^93^ Commonly used oxidizing agents, such as permanganate, chromium-based reagents, and molecular oxygen, allow for the selective oxidation of alcohols to aldehydes or ketones, and further to carboxylic acids. Such transformations are vital for constructing diverse molecular architectures and enhancing functionality in target compounds. In addition to their synthetic utility, oxidation reactions are crucial in biochemical processes.^94^ Metabolic pathways, such as cellular respiration, rely heavily on oxidation–reduction reactions to convert organic substrates into energy. For instance, glucose oxidation in glycolysis and the citric acid cycle leads to the production of adenosine triphosphate (ATP), the energy currency of cells.^95^ The intricate mechanisms of biological oxidation not only facilitate energy transfer but also maintain the balance of redox states in living organisms, proving the vital nature of oxidation reactions in sustaining life. In conclusion, oxidation reactions serve as a cornerstone of organic chemistry, underpinning synthetic strategies, biological functions, and environmental chemistry.^96^ Their significance extends beyond mere chemical transformations; they are essential for innovation in chemical synthesis, the functioning of biological systems, and the mitigation of environmental challenges.^97^ As research continues to evolve, the understanding of oxidation processes will undoubtedly foster advancements in multiple fields, reinforcing their critical role in both academia and industry.

#### 2.1.1. Oxidation of alkynes to epoxides

Epoxides, or oxiranes, are three-membered cyclic ethers characterized by their unique ring structure, which imparts significant reactivity and versatility.^98^ These compounds hold considerable biological, pharmaceutical, and industrial relevance, underscoring their importance across multiple fields. Biologically, epoxides serve as key intermediates in various metabolic pathways. They can exert substantial effects on human health, acting as potential carcinogens or mutagens due to their ability to form covalent bonds with nucleophiles in biological systems.^99^ Furthermore, they participate in the biosynthesis of numerous natural products, emphasizing their role in the chemistry of life. Pharmaceutically, epoxides are pivotal in the development of drugs. Many synthetic pharmaceuticals utilize epoxide functionalities for enhancing biological activity and selectivity. Notable examples include epoxide-containing antibiotics and anti-cancer agents, where the reactivity of the epoxide moiety can lead to desired biological outcomes through targeted interactions with cellular components.^100^ Industrial applications of epoxides are vast, particularly in the production of polymers and resins. Epoxy resins, derived from epoxides, are crucial in coatings, adhesives, and composites due to their excellent mechanical properties and resistance to chemicals and heat. Their utility is also prominent in the manufacture of surfactants and agrochemicals, demonstrating the versatility of epoxides in various industrial sectors.^101^ The synthesis of epoxides is often achieved through the oxidation of alkenes; however, a more efficient and strategically significant method involves the oxidation of alkynes.^102^ Alkynes, with their triple bond, can be selectively oxidized to yield epoxides, thereby harnessing their inherent reactivity. This process typically involves the intermediate formation of ketenes, which can subsequently react with electrophiles or undergo cyclization to produce the desired epoxides.^103^ This methodology not only affords high yields but also offers the possibility of functional group transformations within the same synthetic route, resulting in a streamlined approach to epoxide synthesis. Moreover, the regioselectivity and stereochemistry of the resulting epoxides can be precisely controlled, making this method highly desirable in synthetic organic chemistry.

**Scheme 1:**
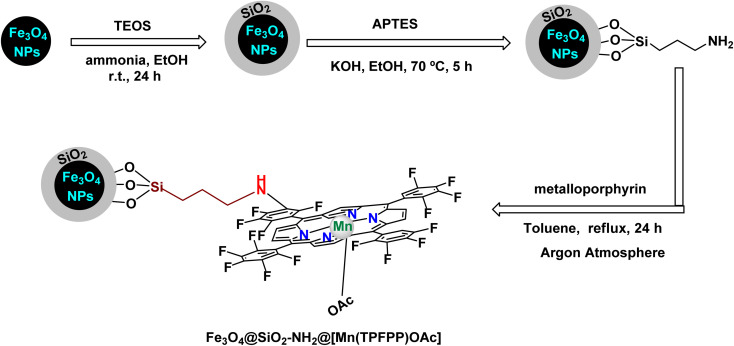
General method to construct the Fe_3_O_4_@SiO_2_–NH_2_@[Mn(TPFPP)OAc] nanocomposite.

**Scheme 2:**
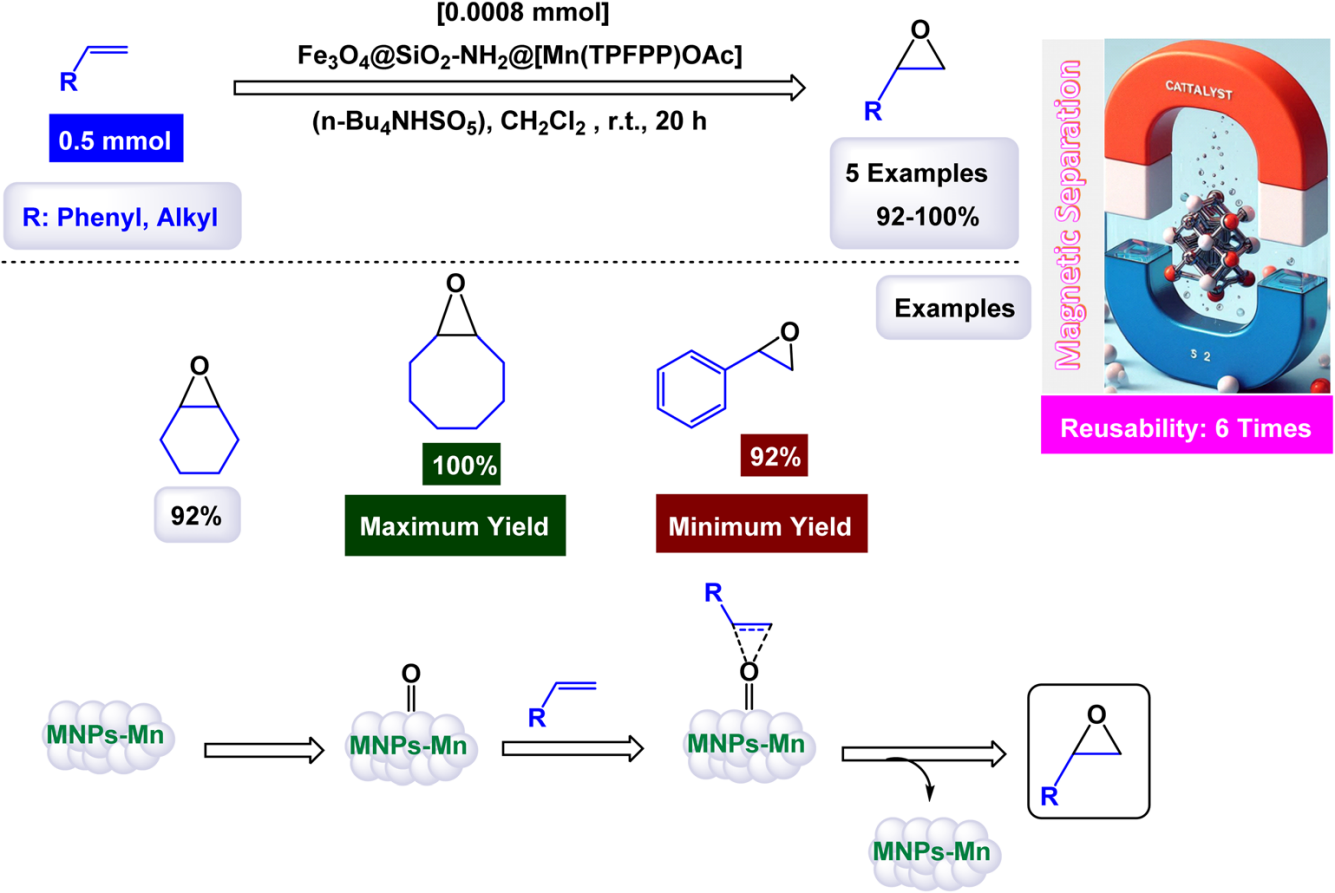
Epoxidation of alkenes [catalysis by Fe_3_O_4_@SiO_2_–NH_2_@[Mn(TPFPP)OAc] nanocomposite].

To construct the magnetically recoverable catalyst based on manganese for catalysis, Mn(TPFPP)OAc, porphyrin, meso-tetrakis(pentafluorophenyl)porphyrin, and manganese(iii) acetate were functionalized with 3-aminopropyltriethoxysilane (APTS) *via* aminopropyl linkage on silica-coated Fe_3_O_4_ magnetic nanoparticles.^104^ This allowed the production of Fe_3_O_4_@SiO_2_–NH_2_@MnPor catalyst by covalently immobilizing Mn(iii) porphyrin *via* aromatic nucleophilic substitution to afford Fe_3_O_4_@SiO_2_–NH_2_@MnPor catalyst as shown in the Scheme 1. By Mojtaba Bagherzadeh and co-workers^104^ and used as a heterogeneous catalyst in the oxidation of alkanes and alkenes. Characterized by different characterization tools, the XRD pattern of the manganese-supported catalyst showed characteristic peaks and relative intensities consistent with standard samples. In the scanning electron microscope analysis, the particle distribution is uniform with an average size of 27 nm, by analyzing the FT-IR spectra, absorption bands at 759, 941, 987, 1492, 1517, and 1649 cm^−1^ were observed which are due to the metal-porphyrin complex and are consistent with the UV-Vis spectroscopy, which showed Soret bands and Q bands at 475 and 576 nm after the immobilization of the metal-porphyrin complex. The activity of the catalyst in oxidation reactions was studied, first, alkane oxidation was carried out using different oxidants *n*-butylammonium monohydrosulfate tetrahydrofuranate (*n*-Bu_4_NHSO_5_) and iodosylbenzene (PhIO) dichloromethane medium in the presence of (8 × 10^−4^, 297 mmol) of catalyst for 20 h where *n*-Bu_4_NHSO_5_ gives the higher oxidation conversion (Scheme 2). The Fe_3_O_4_@SiO_2_–NH_2_@[Mn(TPFPP)OAc] catalyst was also the good efficiency in alkane hydroxylation reactions. The Fe_3_O_4_@SiO_2_–NH_2_@[Mn(TPFPP)OAc] catalyst was re-dissociated in the epoxidation of cyclooctene as a model reaction and used 6 times without a significant decrease in its activity.

**Scheme 3:**
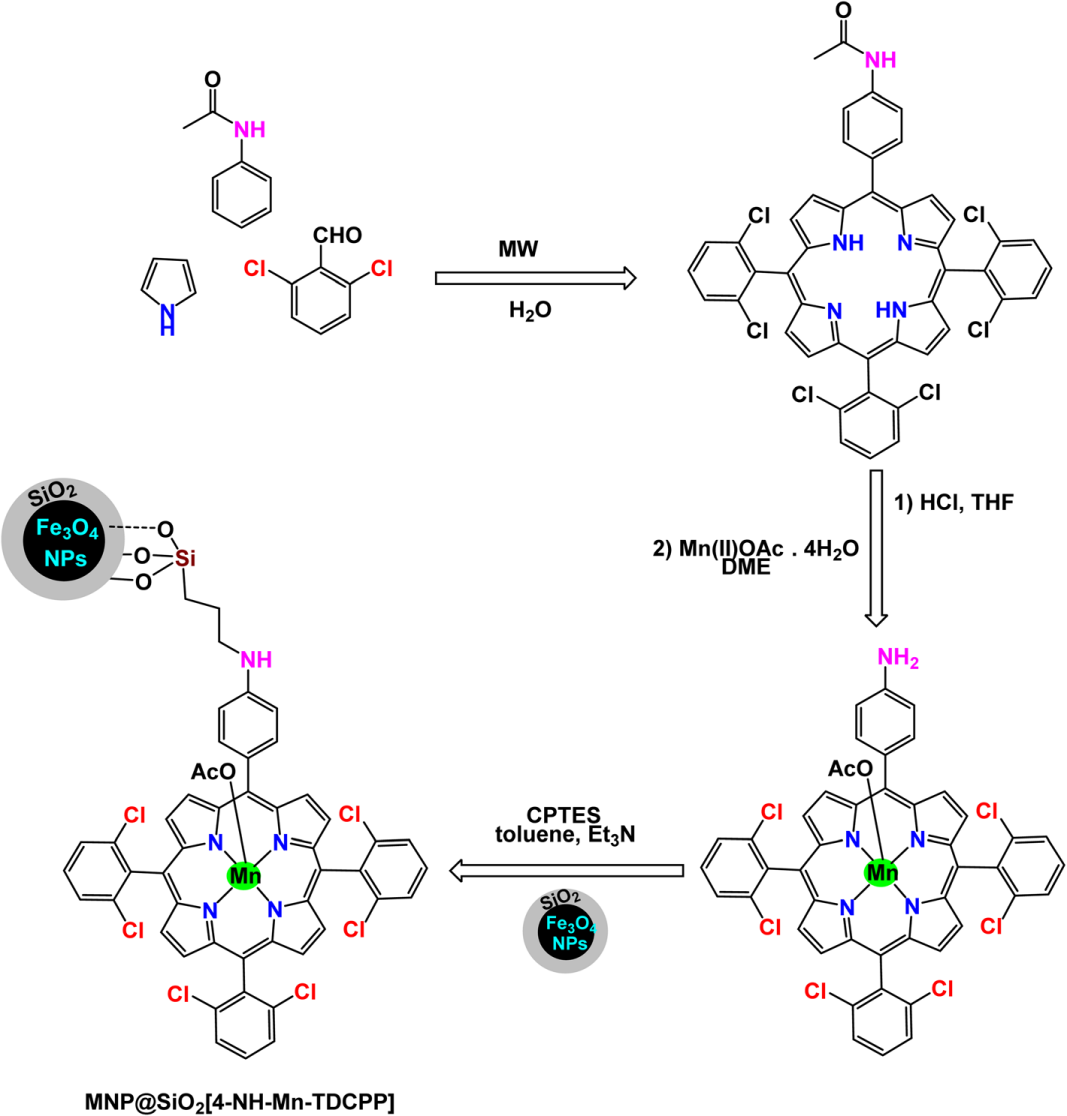
General method to construct the MNP@SiO_2_[4-NH-Mn-TDCPP] nanocomposite.

**Scheme 4:**
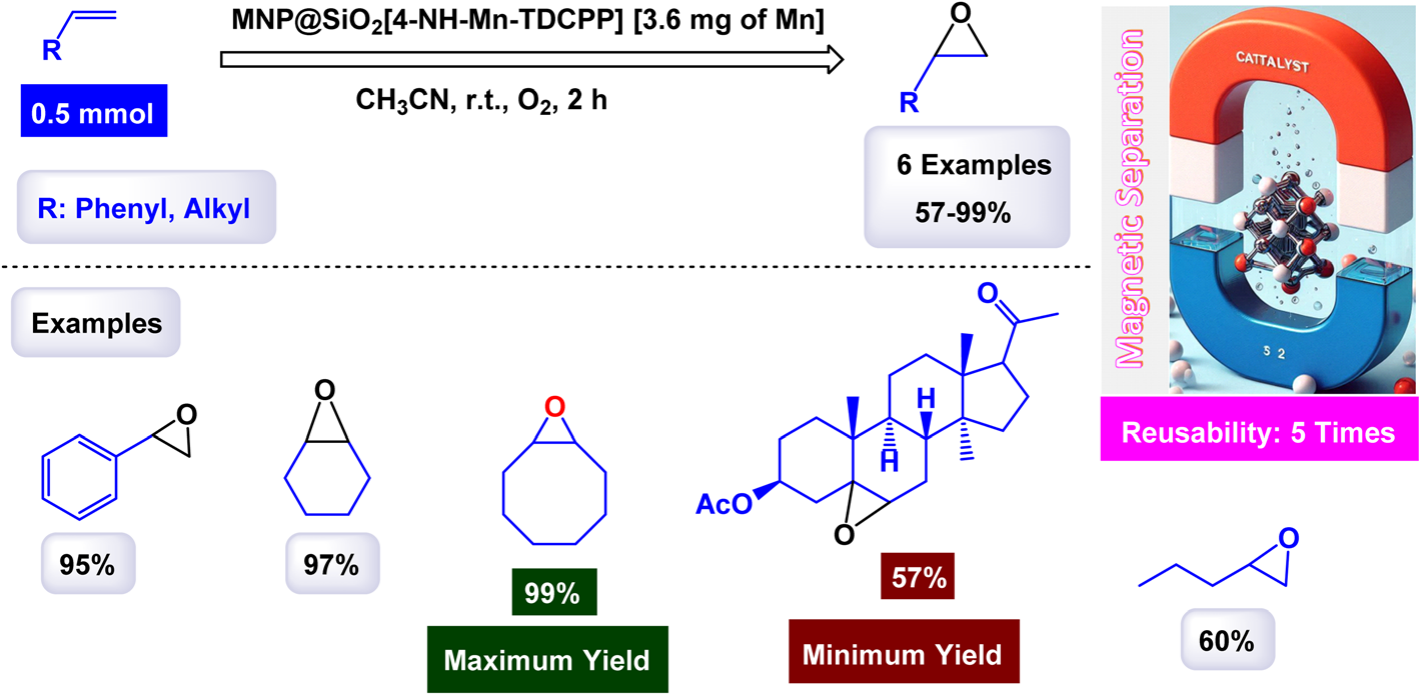
Epoxidation of alkynes [catalysis by MNP@SiO_2_[4-NH-Mn-TDCPP] nanocomposite].

Mariette M. Pereira *et al.* prepared a hybrid magnetic nanocatalyst based on Mn(iii) porphyrin-based by the steps described in the Scheme 3 for the preparation of MNP@SiO_2_[4-NH-Mn-TDCPP].^105^ First, meso-tetraarylporphyrin was prepared, and then in the presence of Mn(OAc)_2_·4H_2_O, porphyrinatomanganese was obtained. Then, in a single step, 3-chloropropyltriethoxysilane (CPTES) ligand was added with silica-coated magnetic iron ferrite to the porphyrina to manganese to form the catalyst MNP@SiO_2_[4-NH-Mn-TDCPP]. The structure of the catalyst was confirmed by different characterization tools, by UV-vis absorption spectrum analysis, an absorption spectrum at 465 nm was obtained for the catalyst which is similar to that of immobilized metalloporphyrin. The FTIR spectrum shows several characteristic peaks confirming the presence of metalloporphyrin. The catalytic oxidation test was carried out using cyclooctene as a model substrate, molecular oxygen as an oxidant, and aldehyde as a co-reductant, in the presence of a homogeneous catalyst Mn(iii)–OAc–TDCPP and a magnetic hybrid catalyst MNP@SiO_2_[4-NH-Mn-TDCPP] in the same amount (4.7 × 10^−5^ mmol) at room temperature for 1.5 h (Scheme 4). Initially, the oxidation reaction was tested without a catalyst and there were no yields. Mn(iii)–OAc–TDCPP–NH_2_ was tested as a homogeneous catalyst and gave excellent yields of 98%, but an increase in conversion values was observed in the first 30 min of the reaction (up to 70%) after that it was slower indicating the catalyst decomposition, which was confirmed by UV-Vis analysis. As for the heterogeneous catalyst, it gave excellent yields of 99% with high stability and no decomposition in addition to the possibility of promising separation and simple recycling. The magnetic MNP@SiO_2_[4-NH-Mn-TDCPP] catalyst exhibited remarkable stability in cyclooctene oxidation, as it maintained both catalytic activity and selectivity through five consecutive reuse cycles. This performance underscores its robustness and efficiency in the reaction.

**Scheme 5:**
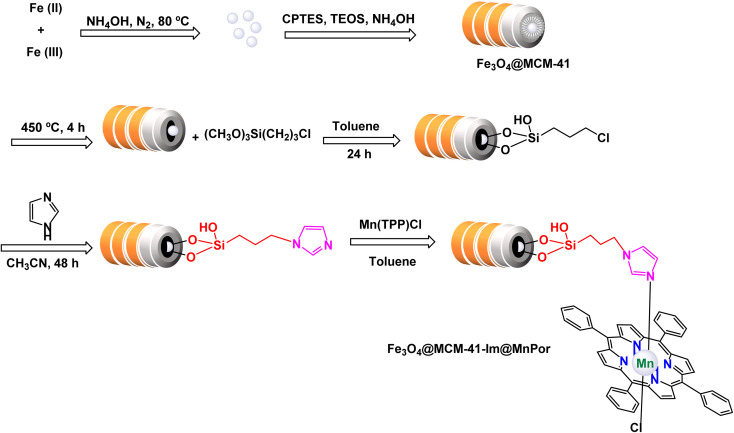
General method to construct the Fe_3_O_4_@MCM-41-Im@MnPor nanocomposite.

**Scheme 6:**
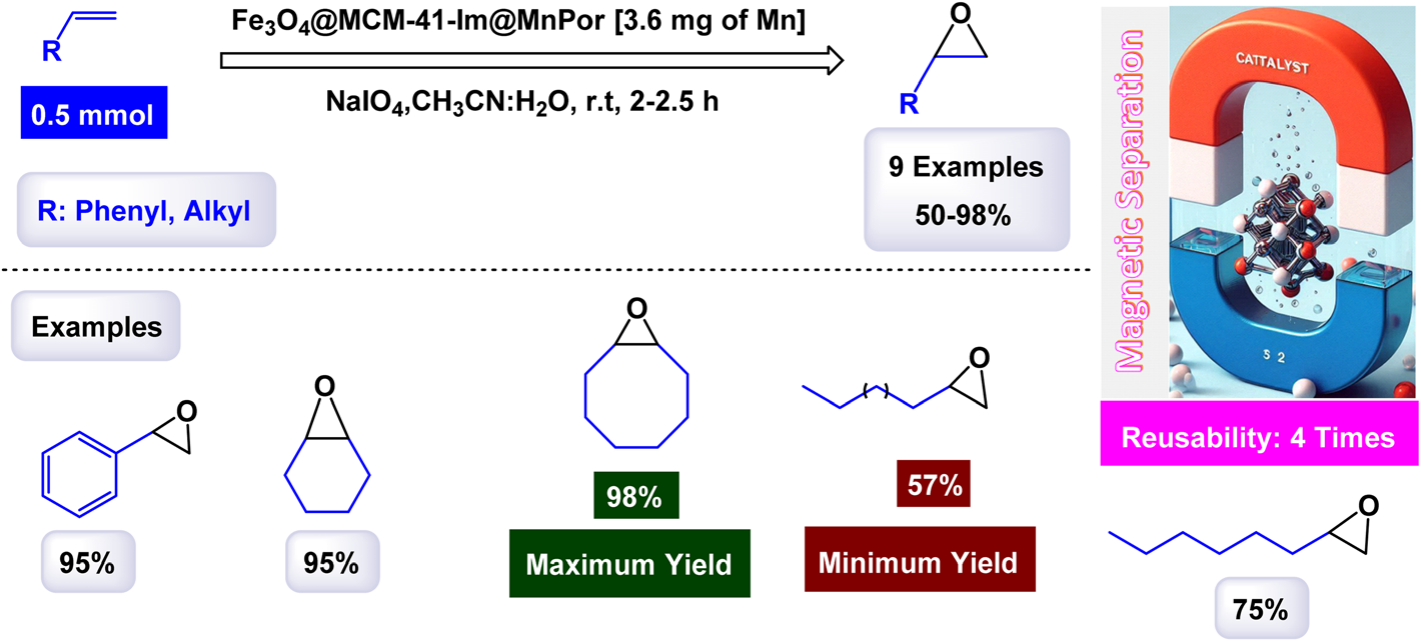
Epoxidation of alkynes [catalysis by Fe_3_O_4_@MCM-41-Im@MnPor nanocomposite].

In a study conducted by Hajian, the immobilization of tetraphenyl-porphyrinatomanganese(iii) chloride, (MnPor) on MCM-41 modified with imidazole as a ligand, which contains Fe_3_O_4_@MCM-41-Im core of magnetic nanoparticles to be the final catalyst Fe_3_O_4_@MCM-41-Im@MnPor as shown in the Scheme 5.^106^ The catalyst was identified through different characterization tools, through FT-IR, frequency bands were observed that are related to porphyrin, but due to the low loading of porphyrin, the bands appeared with low intensity, which is confirmed by UV-Vis spectra, where bands were observed indicating the presence of MnPor fixed on the magnetic support. The activity of the catalyst was studied through its use in different epoxidation reactions of olefins. Cyclooctene was chosen as a model reaction and NaIO_4_ was used as an oxygen donor with 0.02 mmol of catalyst in a 2 : 1 mixture of acetonitrile/water as shown in the Scheme 6. The catalyst was reused four times, in the first and second cycles, slight catalyst leakage from the support was observed at 0.64% in the first cycle and 0.50% in the second cycle using AAS spectroscopy but in the remaining cycles, no leakage was observed. The Fe_3_O_4_@MCM-41-Im@MnPor catalyst can be easily recovered with an external magnet and reused up to four times while retaining its activity and magnetic properties, making it a highly efficient and durable option for catalysis.

#### 2.1.2. Oxidation of sulfides to sulfoxides

Sulfoxides, characterized by the presence of a sulfur atom bonded to an oxygen atom and two carbon groups, are significant compounds in various domains, including biology, pharmaceuticals, and industry.^107,108^ Their unique structural features afford them diverse chemical properties, making them valuable across numerous applications.^109,110^ Biologically, sulfoxides serve as crucial intermediates in the metabolism of sulfur-containing compounds. They play essential roles in the synthesis and degradation of biologically active molecules, including certain amino acids and nucleotides.^111^ Notably, sulfoxides can exhibit activity in biological systems, influencing metabolic pathways and cellular signaling. Pharmaceutically, sulfoxides have garnered considerable attention due to their therapeutic potential.^112^ Many sulfoxide-containing compounds have been developed as drugs, exhibiting a range of biological activities, such as anti-inflammatory, antimicrobial, and antitumor effects.^113^ Their ability to enhance drug solubility and permeability makes sulfoxides prevalent in drug formulation and delivery systems, ultimately improving bioavailability and therapeutic efficacy. From an industrial perspective, sulfoxides are utilized as solvents, reagents, and intermediates in organic synthesis. The unique polar nature of sulfoxides makes them effective in facilitating chemical reactions and dissolving a broad spectrum of organic compounds.^114^ They are also employed in the synthesis of polymers and agrochemicals, underscoring their versatility in industrial applications.^115^ The synthesis of sulfoxides typically involves the oxidation of sulfides, a method that offers both efficiency and selectivity. Sulfides can be readily oxidized using various oxidizing agents such as hydrogen peroxide. This transformation is significant due to the mild reaction conditions needed and its ability to proceed with minimal by-products.^116^ The oxidation process not only streamlines the production of sulfoxides but also allows for the introduction of functional groups that can be further manipulated in synthetic chemistry.

**Scheme 7:**
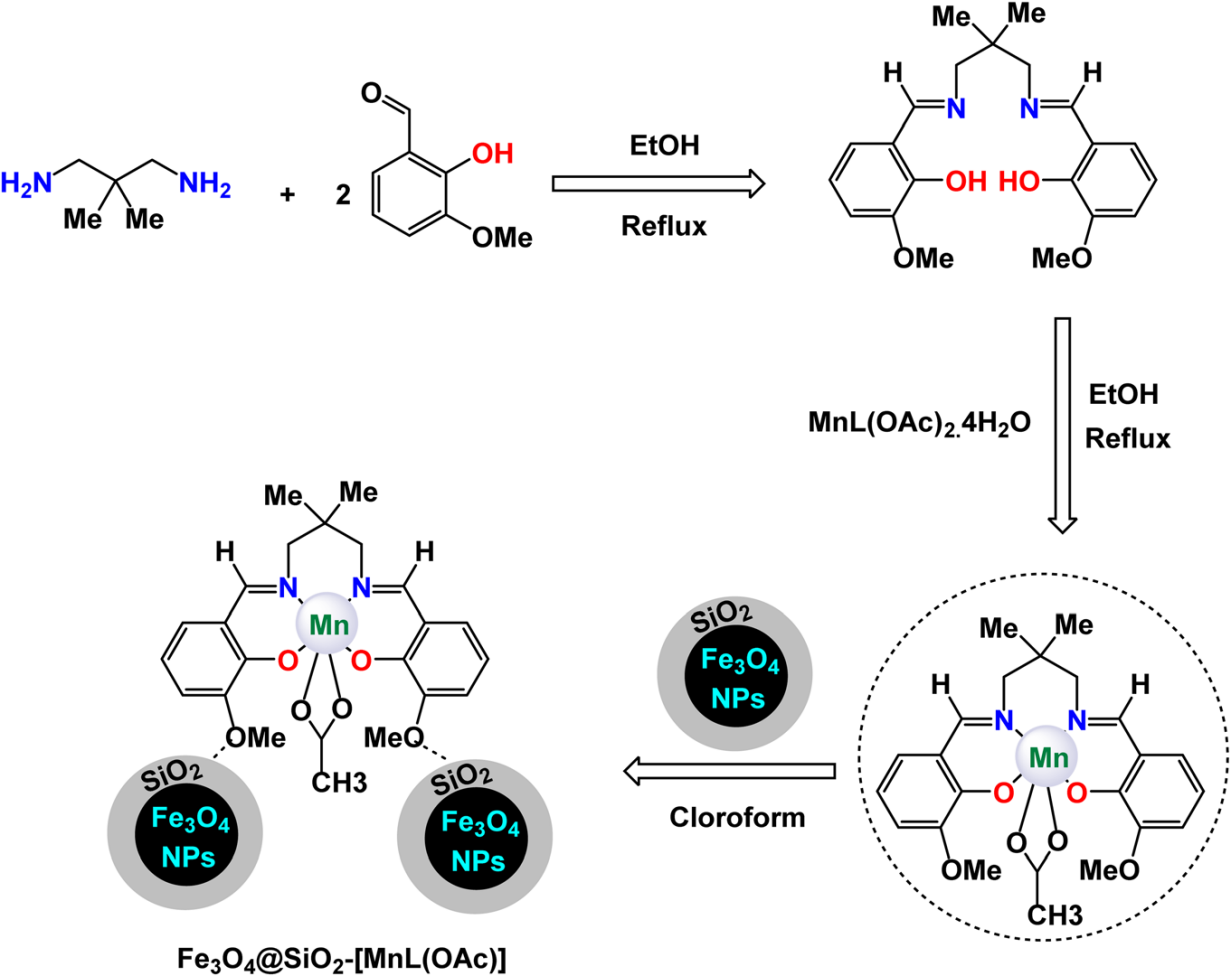
General method to construct the Fe_3_O_4_@SiO_2_–[MnL(OAc)] nanocomposite.

**Scheme 8:**
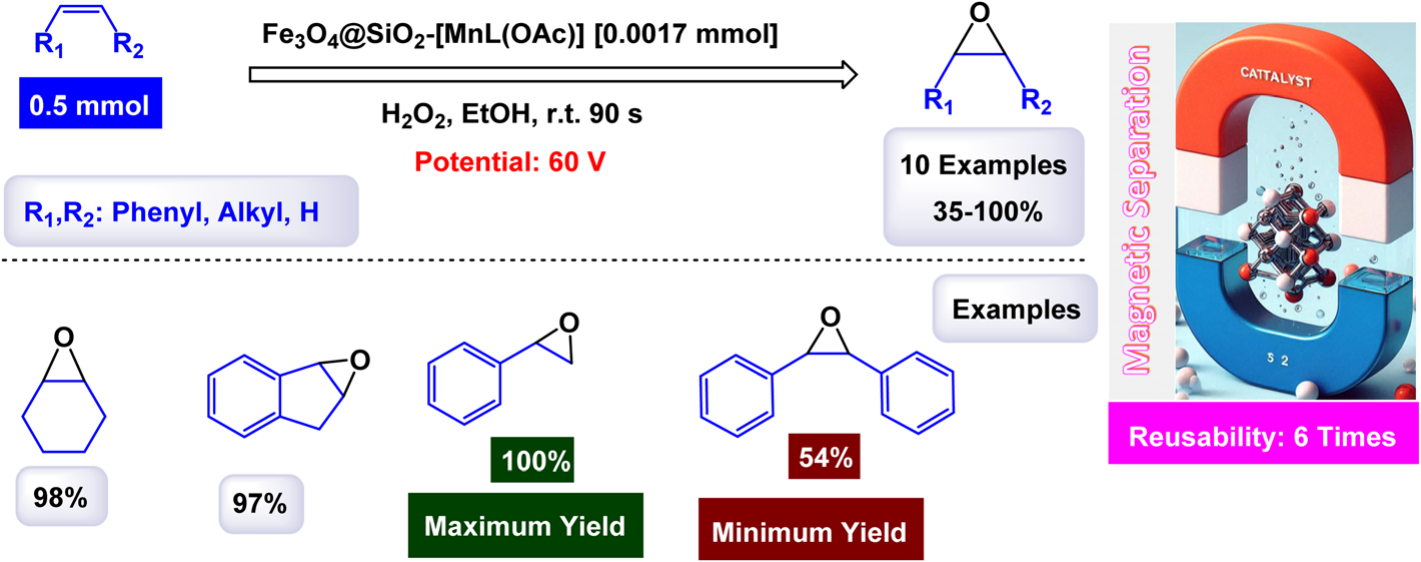
Epoxidation of alkynes [catalysis by Fe_3_O_4_@SiO_2_–[MnL(OAc)] nanocomposite].

**Scheme 9:**
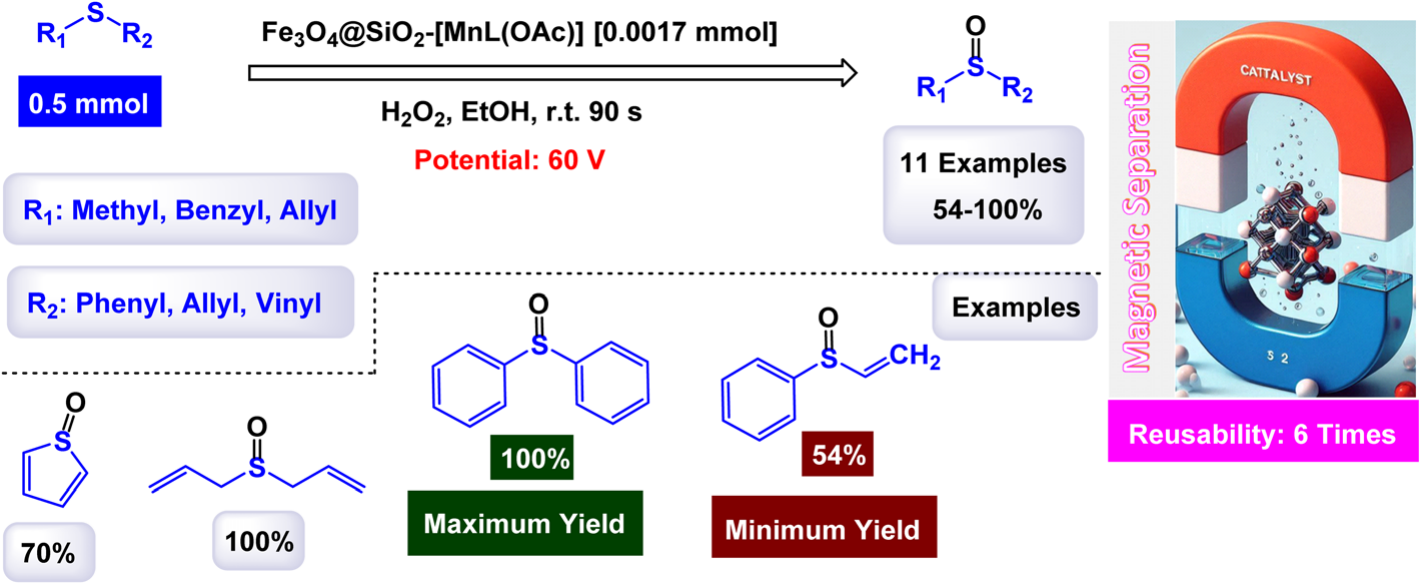
Oxidation of sulfides to sulfoxides by H_2_O_2_ [catalysis by Fe_3_O_4_@SiO_2_–[MnL(OAc)] nanocomposite].

Rayati and his co-workers developed a novel Mn(iii) scaffold supported on silica-coated magnetic iron(iii) oxide *via* one-step axial coordination to form the final product Fe_3_O_4_@SiO_2_–[MnL(OAc)] as shown in the Scheme 7. The heterogeneous nanocatalyst was identified by different characterization tools, FTIR spectra showed an absorption band at 480 cm^−1^ due to Mn–O bond and an absorption band at 1600 and 1650 cm^−1^ due to the C=N group, which can be attributed to the synergy of nitrogen atom in azomethine group with manganese metal. SEM and TEM images showed a smooth spherical shape after Fe_3_O_4_ encapsulation and [MnL(OAc)] immobilization *via* axial coordination. EDX analysis confirmed the presence of all elements that make up the final product, which confirms the purity of the magnetic nanocatalyst.^117^ Also, a decrease in the magnetic saturation value from 62.21, 49.67, and 30.63 was observed due to the encapsulation process and stabilization of the Mn–Schiff base complex *via* axial coordination by VSM analysis. By X-ray diffraction, the catalyst showed its compatibility with the standard sample of Fe_3_O_4_, confirming the absence of deformation of the Fe_3_O_4_ core and the average size of 54.5 nm for Fe_3_O_4_@SiO_2_–[MnL(OAc)]. The activity of the catalyst was tested in the electrochemical oxidation of methyl phenyl sulfide and cyclooctene using H_2_O_2_ in the presence of a glassy carbon electrode as shown in the schematic. In the oxidation of methyl phenyl sulfide, it was carried out at room temperature by the reaction of imidazole, MePhS, and H_2_O_2_ in ethanol with 0.0017 mmol of catalyst as standard conditions, in the oxidation of cyclooctene, the same conditions were used in the presence of cyclooctene. To obtain the best reaction conditions, the reaction was started without a catalyst, and in the presence of Fe_3_O_4_@SiO_2_, there were no yields within 90 seconds. However, in the presence of [MnL(OAc)] unsupported, 95% yields were obtained. Using the Fe_3_O_4_@SiO_2_–[MnL(OAc)] catalyst, almost the same yields were obtained within the same time of 90 s. Under the standardized conditions presented in Schemes 8 and 9, the oxidation reaction were successfully preformed ant the target oxidized products were obtained with satisfactory yields. The Fe_3_O_4_@SiO_2_–[MnL(OAc)] catalyst was preferred due to its ease of separation and recycling. The Fe_3_O_4_@SiO_2_–[MnL(OAc] catalyst was subjected to a series of tests to evaluate its ability to be reused in the electrochemical oxidation processes of methylphenyl sulfide and cyclooctene. Remarkably, it maintained consistent catalytic activity throughout six consecutive cycles of operation, demonstrating no degradation in performance or loss of efficiency during the reactions. This indicates a strong resilience and stability of the catalyst under the experimental conditions.

**Scheme 10:**
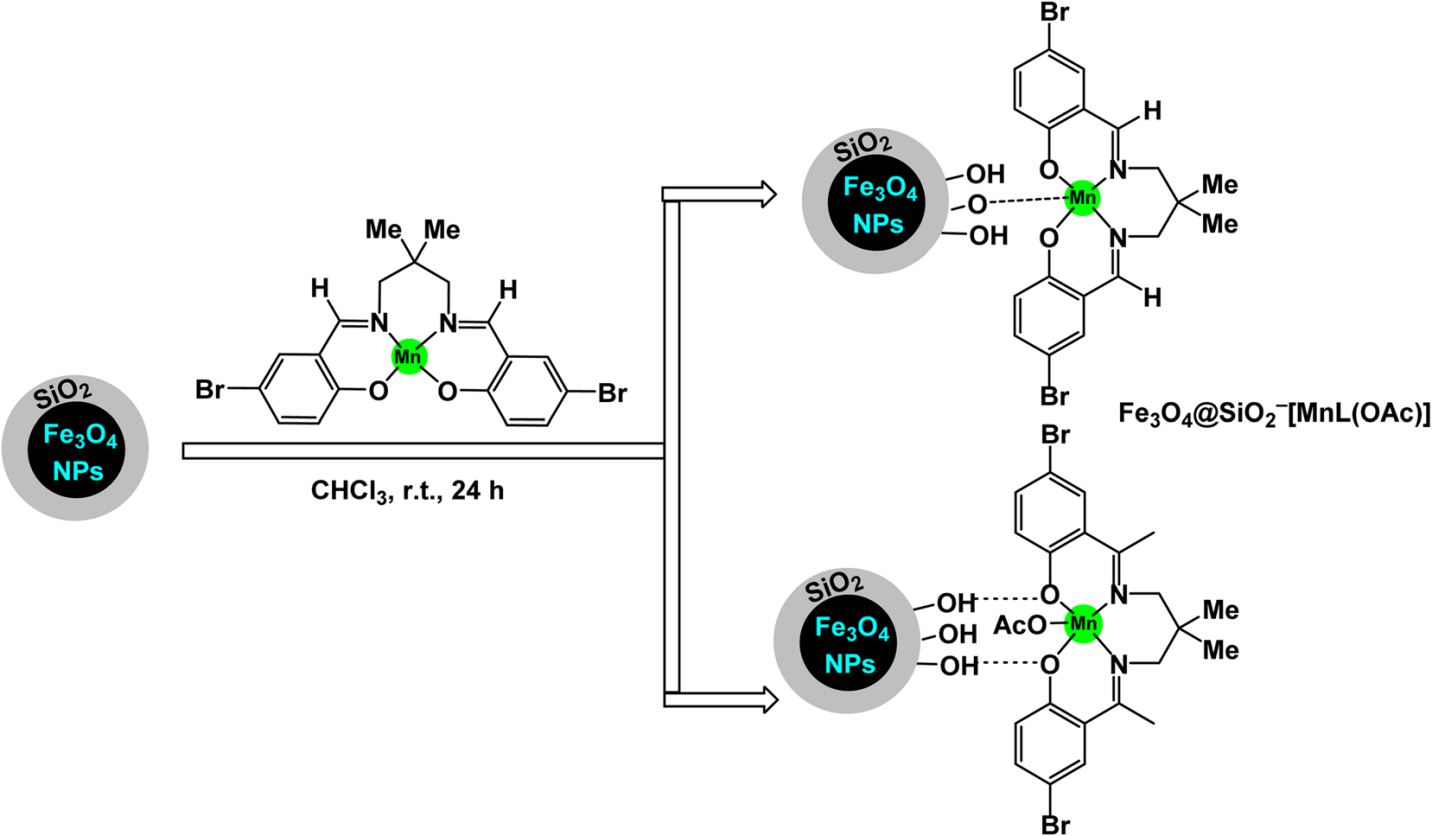
General method to construct the Fe_3_O_4_@SiO_2_–[MnL(OAc)] nanocomposite.

**Scheme 11:**
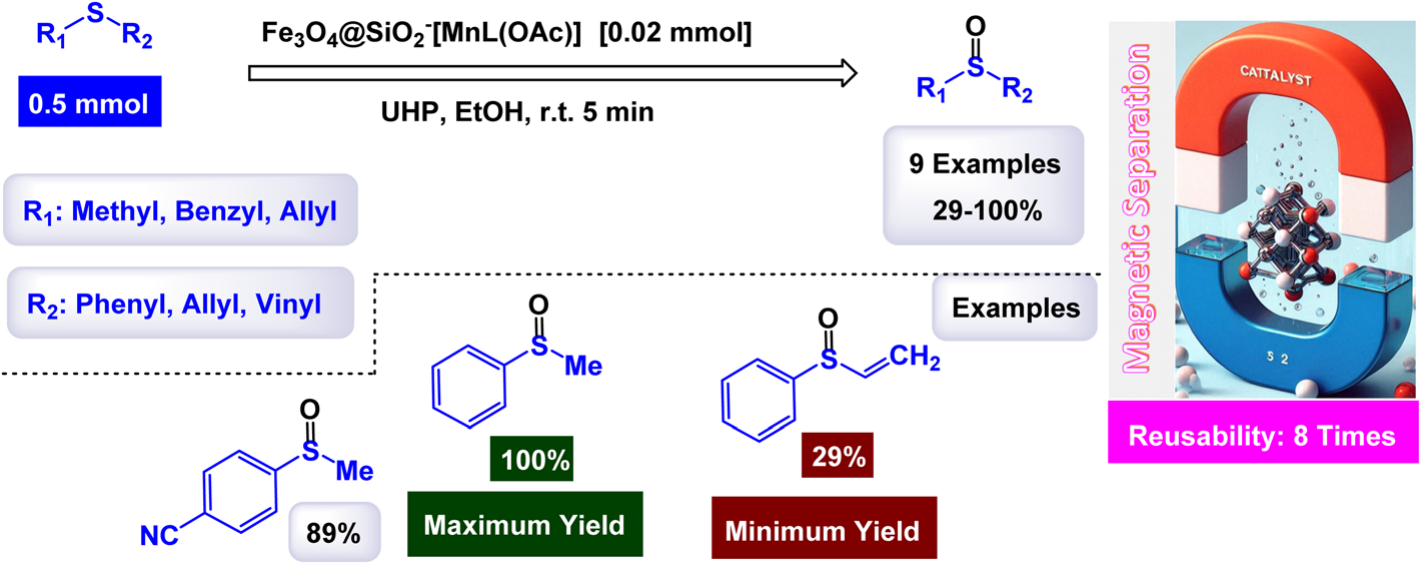
Oxidation of sulfides to sulfoxides by UHP [catalysis by Fe_3_O_4_@SiO_2_–[MnL(OAc)] nanocomposite].

A magnetically recoverable manganese–Schiff base catalyst was prepared by Rayati and his co-workers^117^ by adsorption on magnetic nanoparticles Fe_3_O_4_@SiO_2_ to form Fe_3_O_4_@SiO_2_–[MnL(OAc)] as shown in the Scheme 10. Transmission electron microscopy TEM analysis shows the spherical shape of the particles and their size within the nano range. VSM analysis showed a decrease in the magnetic saturation value due to the formation of layers on the magnetic particle Fe_3_O_4_, which is an important part of proving the successful encapsulation process. FTIR analysis showed vibration bands due to the manganese–Schiff base. The catalyst was tested in the catalytic oxidation of sulfides in the presence of urea–hydrogen peroxide as an oxygen source (Scheme 11), methylphenyl sulfide was used as a model oxidation reaction, imidazole as a reagent, and Fe_3_O_4_@SiO_2_–[MnL(OAc)] as a catalyst in ethanol. This protocol results in excellent yields (100%) and selectivity (100%) within 5 min at room temperature.^118^ The catalyst was tested in the oxidation of cyclooctene under the same conditions for 4 h. By comparing MnL(OAc) and Fe_3_O_4_@SiO_2_–[MnL(OAc)], higher yields were achieved in the presence of Fe_3_O_4_@SiO_2_–[MnL(OAc)] than in [MnL(OAc)], confirming the superiority of the catalyst supported on magnetic nanoparticles over the homogeneous catalyst. The versatile Fe_3_O_4_@SiO_2_–[MnL(OAc)] catalyst demonstrated remarkable durability, maintaining its activity and selectivity over eight consecutive reuse cycles. This consistency highlights its exceptional stability and efficiency as a nanocatalyst.

**Scheme 12:**
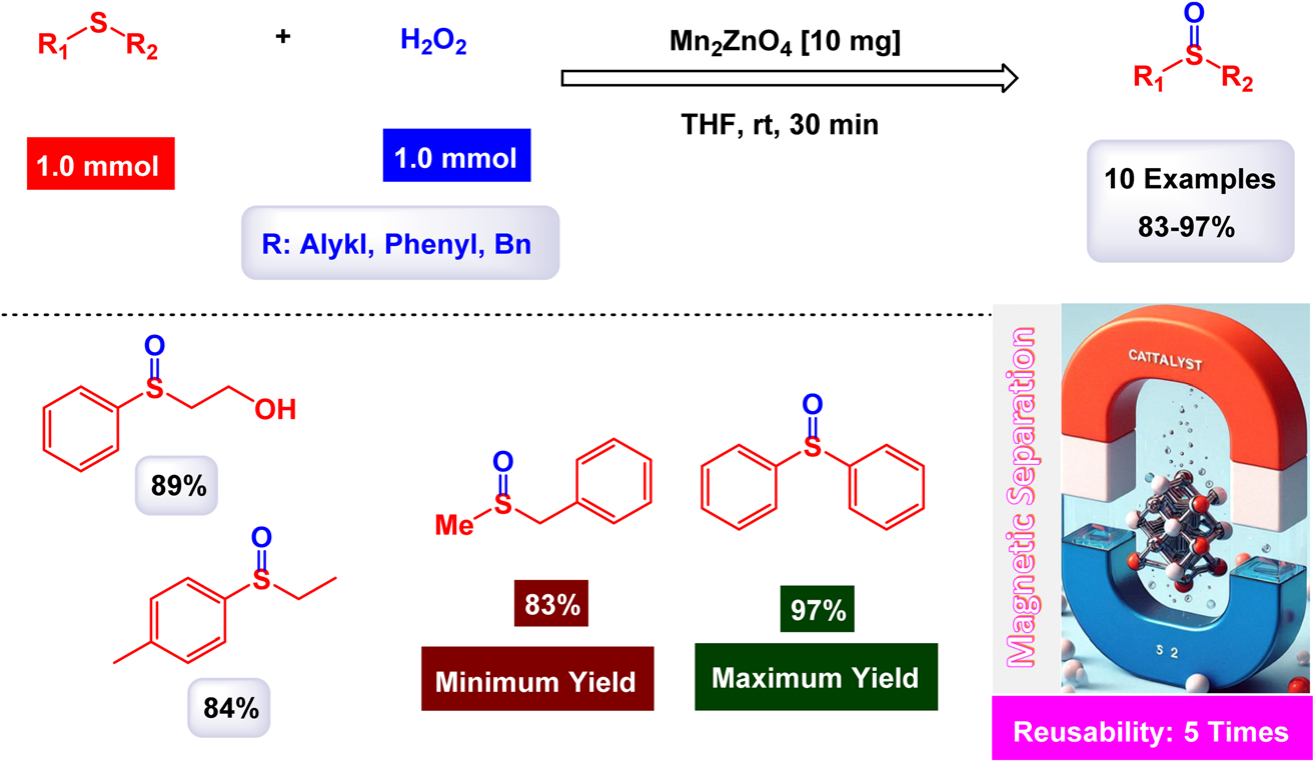
Oxidation of sulfides to sulfoxides by H_2_O_2_ [catalysis by Mn_2_ZnO_4_] nanocomposite].

Gupta and Shroff have meticulously documented the intriguing phenomenon of sulfides undergoing oxidation in the presence of hydrogen peroxide, a process that is magnificently catalyzed by the remarkable MnFe_2_O_4_ nanoparticles, which have shown great potential in enhancing reaction efficiency.^110^ The synthesis of the Mn_2_ZnO_4_ spinel nanocomposite was accomplished through the intricate sol–gel method, utilizing oxalic acid as a chelating agent, and this process was carried out at impressively low temperatures to ensure optimal formation of the nanostructures. The detailed TEM images reveal that the nanoparticles are uniformly shaped spherical crystals, boasting an average diameter of approximately 15 nm, a finding that is corroborated by the analyses conducted using the BET method for surface area evaluation. As illustrated in Scheme 12, a plethora of both aliphatic and aromatic sulfides, each possessing a diverse array of functional groups, can be effectively transformed into their corresponding sulfoxides with remarkably high yields when subjected to the precisely defined reaction conditions that have been set forth. The MnFe_2_O_4_ nanocatalyst demonstrated impressive stability in recycling tests, maintaining its catalytic efficiency with only a slight decline after 5 uses.

#### 2.1.3. Oxidation of thiols to disulfides

Disulfides play a crucial role in medicinal and biological chemistry, as they are essential for stabilizing protein structures and facilitating enzyme activity.^119,120^ The formation of disulfide bonds, which occur between the sulfur atoms of two cysteine residues, contributes to the three-dimensional conformation of proteins, influencing their functionality and interactions.^121,122^ The oxidation of thiols to disulfides involves the removal of hydrogen atoms from two thiol groups (R-SH), resulting in the covalent linkage of the sulfur atoms (R-S-S-R) and the release of water.^120,123^ This biochemical reaction is not only vital for maintaining cellular redox homeostasis but also acts as a regulatory mechanism in various biological processes, including signal transduction and protein folding.^120^

**Scheme 13:**
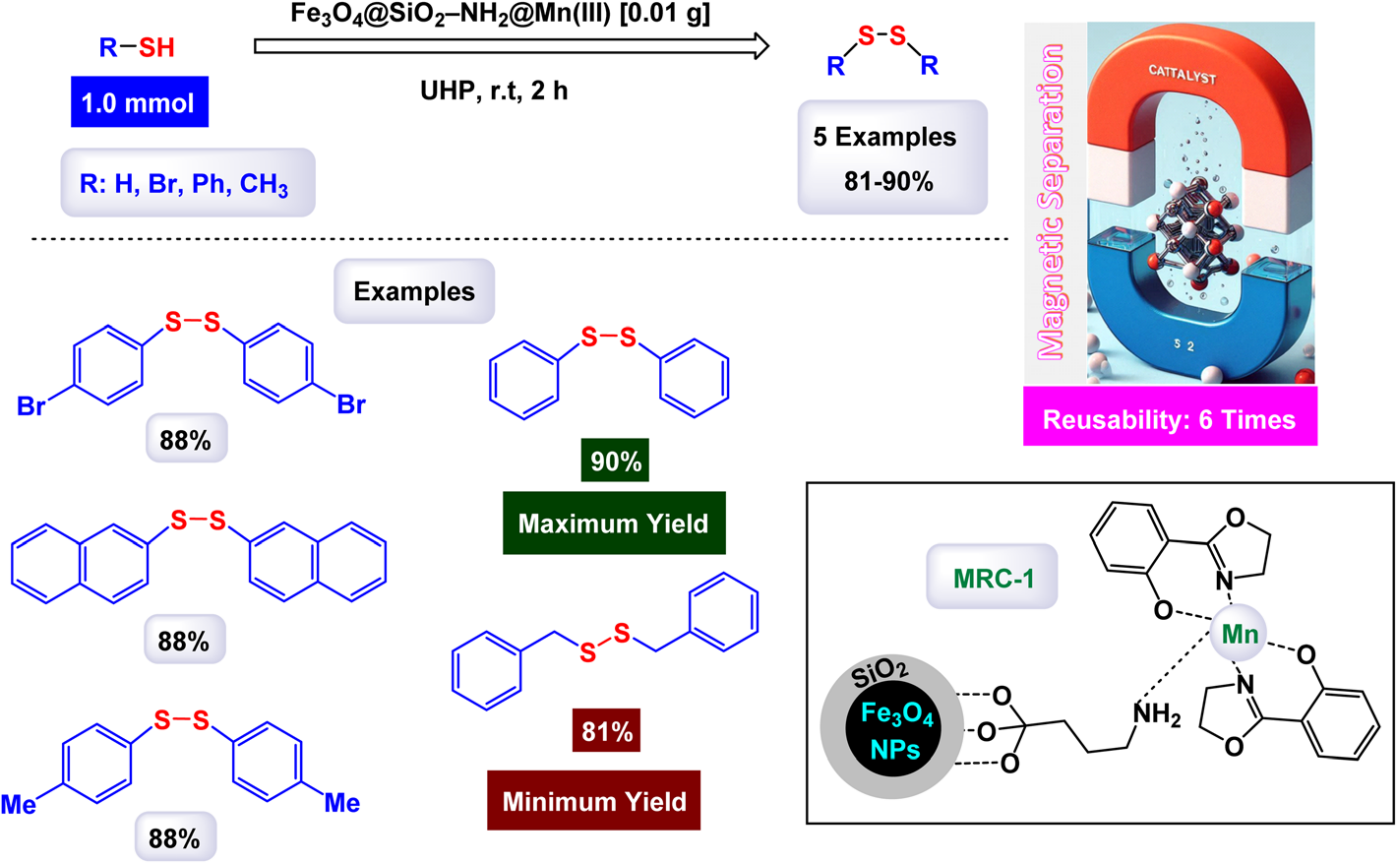
Oxidation thiols to disulfides [catalysis by Fe_3_O_4_@SiO_2_–NH_2_@Mn(iii) nanocomposite].

In a study conducted by Bagherzadeh and his research team, it was reported that manganese immobilized on magnetic nanoparticles can be used for selective oxidation of thiols to dissolve in the presence of urea-hydrogen peroxide as an oxidant (Scheme 13). First, Fe_3_O_4_ was prepared by co-precipitation method, coated with silica, then doped with aminopropyl, and in the last step, the trivalent manganese complex [Mn(pox)_2_(CH_3_OH)_2_]ClO_4_ was immobilized on the magnetic particle. Through different analytical devices, the resulting nanocatalyst Fe_3_O_4_@SiO_2_–NH_2_@Mn(iii) was described. SEM analysis proved that the resulting particle is within the nano dimension with an average size of less than 50 nm and a rough surface. After coating it with silica and aminopropyl, the size did not change much because the existing silica layer is thin, but there was a transformation in the surface in terms of roughness as it turned into a relatively smooth surface, indicating successful coating. Evidence of the successful immobilization of the Mn(iii) complex on the [Mn(pox)_2_ (CH_3_OH)_2_]ClO_4_ complex where (phox = 2-(2′-hydroxyphenyl)oxazoline), through UV-Vis analysis, a significant increase in the C/N ratio from 3.3 in Fe_3_O_4_@SiO_2_–NH_2_ to 3.7 in Fe_3_O_4_@SiO_2_–NH_2_@Mn(iii), by AAS analysis of Mn(iii) complex the loading ratio was 0.0208 mmol g^−1^. To determine the activity of the catalyst in sulfur–sulfur coupling reaction, thiophenol, and UHP were mixed in the presence of CH_2_Cl_2_/MeOH and 0.01 g of nano catalyst Fe_3_O_4_@SiO_2_–NH_2_@Mn(iii) where it gave yields of 81 to 90% while using Fe_3_O_4_ and Fe_3_O_4_@SiO_2_ and Fe_3_O_4_@SiO_2_–NH_2_ there was no oxidation, and when using manganese complex alone [Mn(pox)_2_ (CH_3_OH)_2_]ClO_4_ the results were 55% with the production of unwanted secondary compounds. The Fe_3_O_4_@SiO_2_–NH_2_@Mn(iii) nanocatalyst demonstrated impressive stability in recycling tests, maintaining its catalytic efficiency with only a slight decline after six uses.^124^

#### 2.1.4. Oxidation of alcohols to aldehydes

The oxidation of alcohols to aldehydes is a crucial transformation in organic chemistry, widely used in the synthesis of fine chemicals, pharmaceuticals, and perfumes.^125^ This reaction is important because aldehydes serve as key intermediates in the production of carboxylic acids, esters, and other valuable compounds.^126^ Selective oxidation of primary alcohols to aldehydes, without further oxidation to carboxylic acids, is essential for preserving the desired functional group. Various oxidizing agents, such as PCC (pyridinium chlorochromate) and Swern oxidation, enable this transformation under controlled conditions.^127^ Understanding and optimizing this oxidation process is vital for efficient industrial and laboratory applications.

**Scheme 14:**
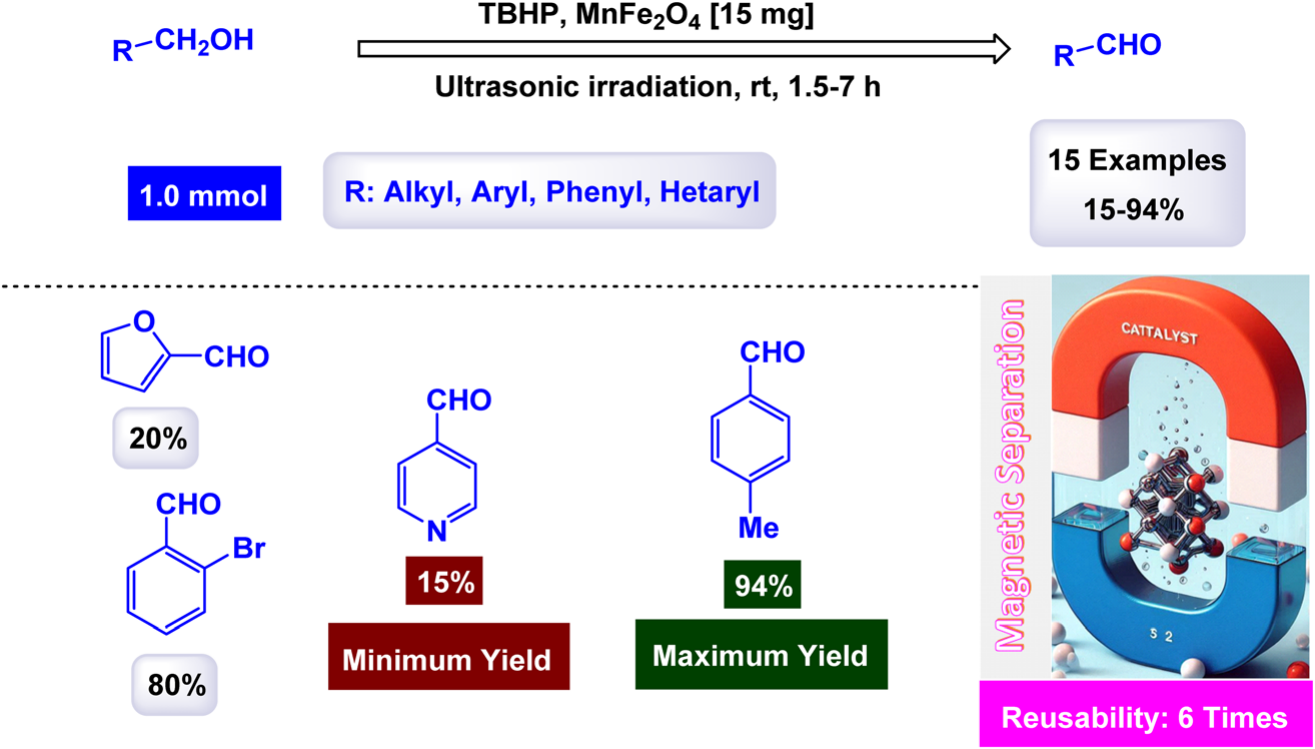
Oxidation selective of benzyl alcohols into benzaldehydes [catalysis by MnFe_2_O_4_ nanocomposite].

**Scheme 15:**
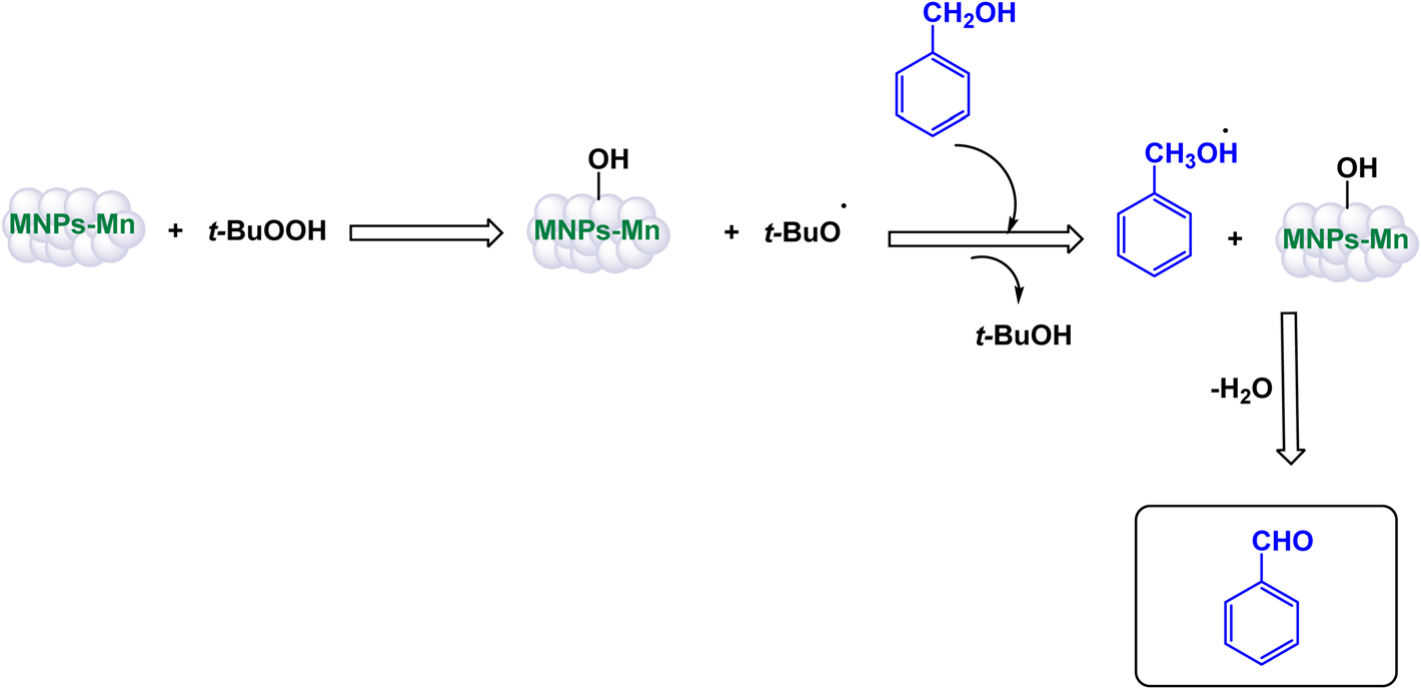
Suggested mechanism for oxidation selective oxidation of benzyl alcohols into benzaldehydes [catalysis by MnFe_2_O_4_ nanocomposite].

Rashid and his research team explored the production of manganese ferrite nanoparticles (MnFe_2_O_4_ NPs) to serve as catalysts for the selective oxidation of benzyl alcohols into benzaldehydes.^128^ X-ray diffraction analysis verified the creation of pure, crystalline MnFe_2_O_4_, with crystallite sizes varying between 18 and 28 nm, based on the additives employed. Scheme 14 demonstrates that every substituted benzyl alcohol was transformed into its related aldehyde with 100% selectivity, showing no additional oxidation to carboxylic acids. The research observed that benzyl alcohols containing electron-donating groups exhibited greater reactivity compared to those with electron-withdrawing groups, emphasizing the influence of substituent electronic effects on the reaction. Drawing from existing literature, they suggest a process for transforming benzyl alcohol into benzaldehyde, as demonstrated in Scheme 15. The procedure starts with the homolytic rupture of TBHP at the O–O bond on the surface of the catalyst, yielding active components *t*-BuOc and hydroxyl radical (HOc). The catalyst interacts with HOc to create intermediate. Subsequently, hydrogen is extracted from benzyl alcohol, resulting in intermediate. The transfer of hydrogen from intermediate to generates benzaldehyde and releases H_2_O as a byproduct, while concurrently restoring the catalyst for subsequent reactions. The MnFe_2_O_4_ nanocatalyst demonstrated impressive stability in recycling tests, maintaining its catalytic efficiency with only a slight decline after 6 uses.

### 2.2. Synthesis of heterocycles

The synthesis of heterocycles is of paramount importance in organic chemistry due to their widespread presence in pharmaceuticals, agrochemicals, and materials science.^129^ Heterocyclic compounds, which contain atoms such as nitrogen, oxygen, or sulfur in their rings, exhibit diverse chemical and biological properties.^130^ Many natural products, including antibiotics, alkaloids, and vitamins, have heterocyclic cores, making their synthetic routes highly valuable. The ability to construct heterocycles efficiently allows chemists to develop new bioactive molecules and optimize existing ones, leading to advancements in drug discovery and therapeutic agents.^131,132^ Among synthetic chemists, heterocycle synthesis is crucial for designing and modifying drug molecules to enhance their pharmacological properties, such as solubility, bioavailability, and target specificity. Many blockbuster drugs, including penicillins, anticancer agents, and antidepressants, are built upon heterocyclic scaffolds.^133^ The ability to introduce functional groups selectively and control stereochemistry in heterocyclic frameworks enables the fine-tuning of biological activity. Moreover, heterocycle synthesis plays a role in overcoming drug resistance by providing novel structural modifications that improve efficacy against diseases. Beyond pharmaceuticals, heterocyclic compounds find applications in materials science, such as in organic semiconductors, dyes, and polymers. Their unique electronic and optical properties make them essential for the development of OLEDs, solar cells, and conductive materials.^134^ Additionally, heterocycles serve as key intermediates in synthetic methodologies, facilitating the construction of complex molecular architectures. As a result, advancements in heterocycle synthesis drive progress across multiple scientific disciplines, reinforcing its significance among synthetic chemists.

#### 2.2.1. Synthesis of imidazole derivatives

Imidazole derivatives are an important class of heterocyclic compounds with significant biological and pharmaceutical properties.^135^ The imidazole ring, consisting of a five-membered system with two nitrogen atoms, is found in many natural biomolecules, including histidine and the related enzyme histamine.^136,137^ Due to its unique electronic and hydrogen bonding properties, imidazole and its derivatives exhibit a wide range of biological activities such as antibacterial, antifungal, antiviral, anti-inflammatory, and anticancer effects.^138,139^ Many pharmaceutical agents, including antifungal drugs like ketoconazole and miconazole, incorporate imidazole derivatives due to their ability to interfere with enzyme systems and microbial cell membranes.^140,141^ Additionally, imidazole-based compounds serve as key components in enzyme inhibitors and proton pump inhibitors, making them crucial in medical and therapeutic applications.^142,143^ Due to these significant applications, the synthesis of imidazole derivatives is of great importance in organic synthesis, as researchers continuously develop new derivatives to improve drug efficacy and reduce side effects.

**Scheme 16:**
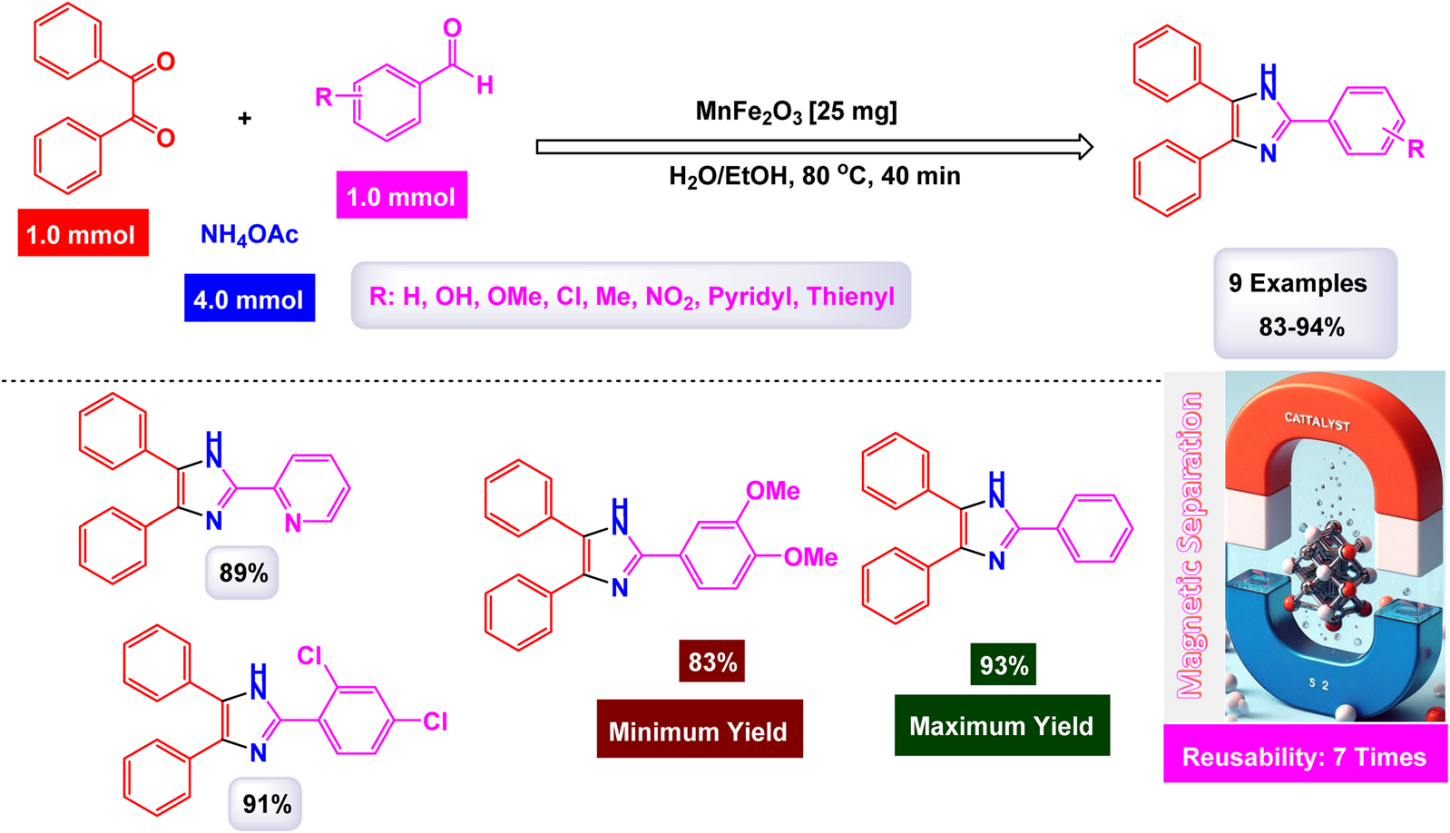
Synthesis of triaryl imidazoles [catalysis by MnFe_2_O_4_ nanocomposite].

In a significant advancement, Mahmudzadeh and his research team meticulously prepared size-controlled MnFe_2_O_4_ nanoparticles through a well-defined chemical co-precipitation technique. They explored the remarkable catalytic performance of these nanoparticles in the synthesis of triaryl imidazole compounds, employing a three-component reaction that combines aromatic aldehydes, benzil, and ammonium acetate in a favorable mixture of water and ethanol (illustrated in Scheme 16).^144^ The TEM images reveal that these nanoparticles possess a nearly spherical shape, exhibiting a uniform size distribution that ranges from 20 to 30 nm, with an average dimension of 23 nm. These size measurements are consistent with those derived from XRD analysis, confirming the structural integrity and uniformity of the particles while highlighting their potential efficacy in catalytic applications. This combination of precise preparation and characterization positions MnFe_2_O_4_ nanoparticles as promising candidates for enhancing synthesis methods in organic chemistry. The MnFe_2_O_4_ nanocatalyst demonstrated impressive stability in recycling tests, maintaining its catalytic efficiency with only a slight decline after 7 uses.

**Scheme 17:**
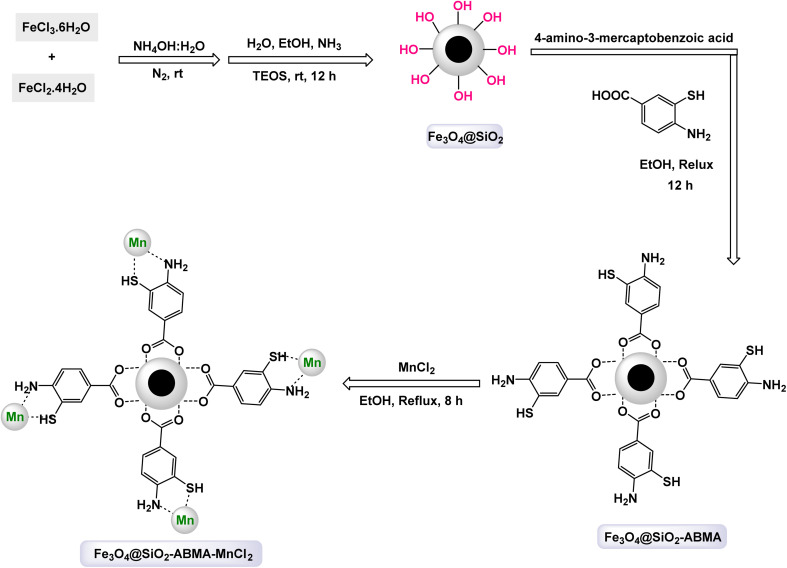
General method for construction of Fe_3_O_4_@SiO_2_–ABMA–MnCl_2_ nanocomposite.

**Scheme 18:**
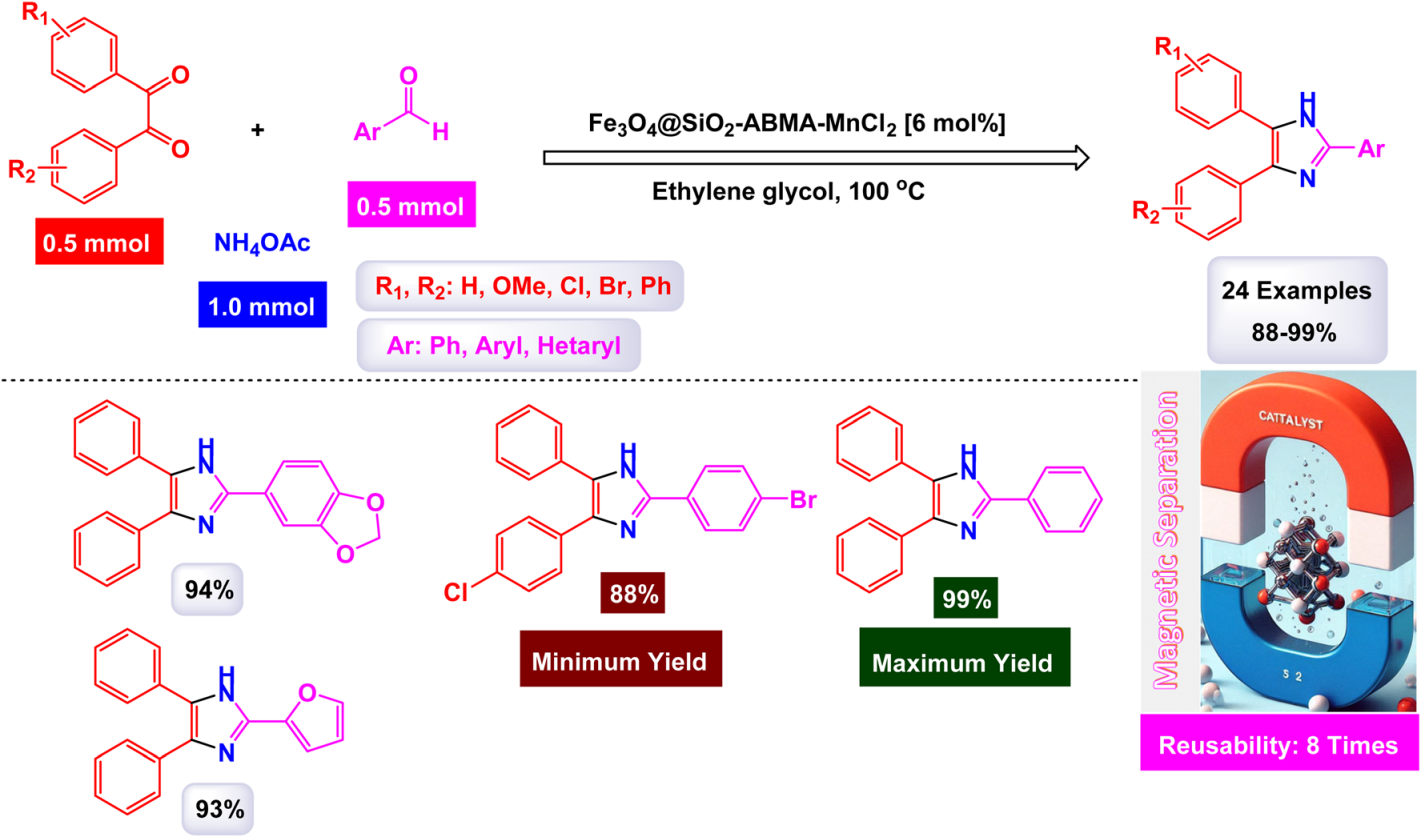
Synthesis of triaryl imidazoles [catalysis by Fe_3_O_4_@SiO_2_–ABMA–MnCl_2_ nanocomposite].

**Scheme 19:**
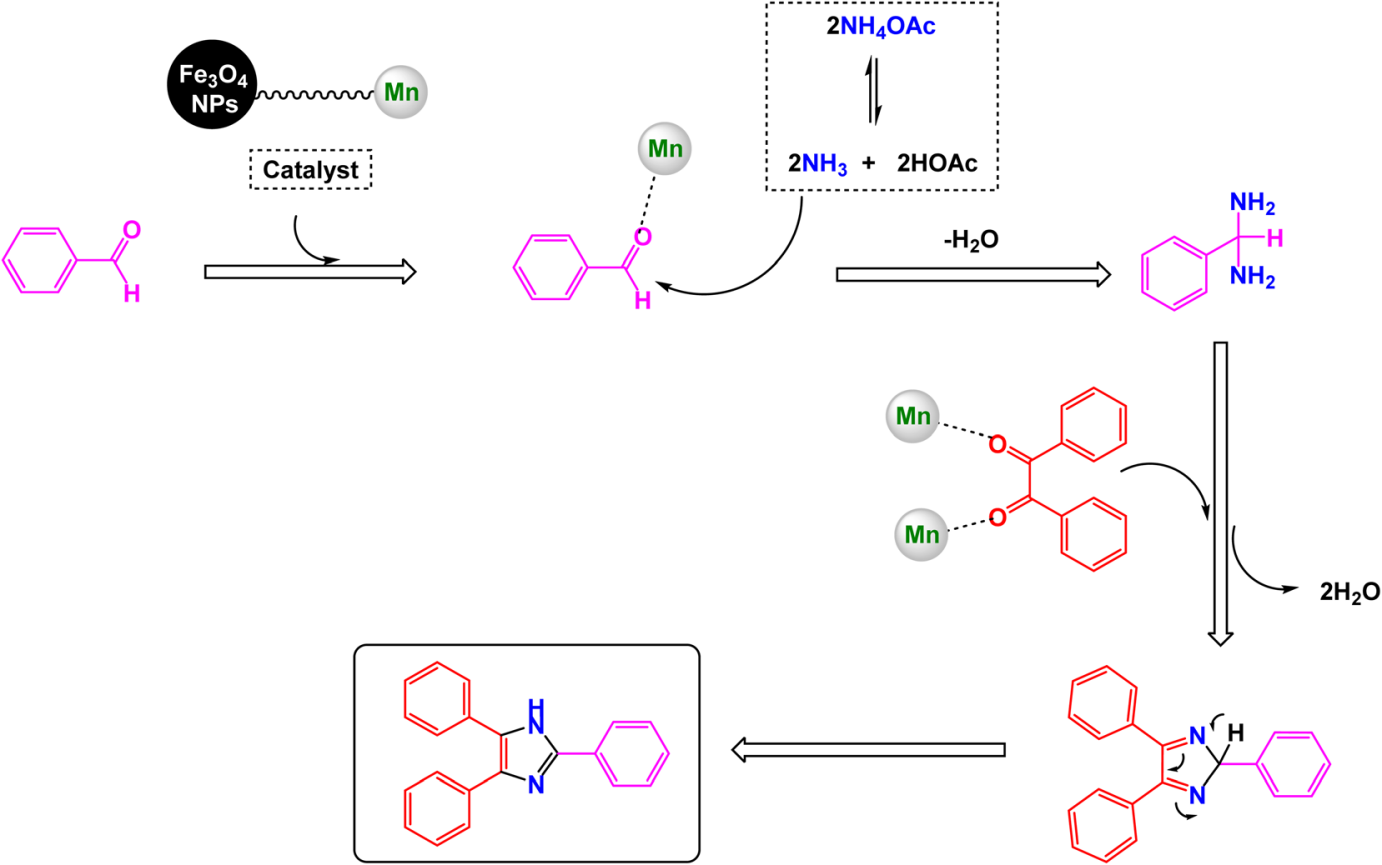
Suggested mechanism for synthesis of triaryl imidazoles [catalysis by Fe_3_O_4_@SiO_2_–ABMA–MnCl_2_ nanocomposite].

In an innovative research, Shafik and his team have engineered a groundbreaking and recoverable nanocatalyst known as Fe_3_O_4_@SiO_2_–ABMA–MnCl_2_, specifically designed for the synthesis of 2,4,5-triaryl imidazoles.^145^ This synthesis is achieved through a dynamic three-component reaction that involves a diverse range of derivatives from aromatic and heteroaromatic aldehydes, along with benzil derivatives and ammonium acetate, conducted in a highly efficient ethylene glycol medium. The meticulous construction of the Fe_3_O_4_@SiO_2_–ABMA–MnCl_2_ nanocomposite is detailed thoroughly in Scheme 17. The process begins with the strategic immobilization of manganese(ii) chloride onto the surface of the Fe_3_O_4_@SiO_2_–ABMA ligand, performed under refluxing conditions in ethanol. This method ensures a successful synthesis of the nanocomposite, leading to a remarkable catalyst with tailored properties. Characterization of the nanocomposite was carried out using SEM and TEM, revealing a uniform distribution of spherical particles throughout the sample. The particle sizes, analyzed through XRD, were confirmed to range from 15 to 30 nanometers, indicating the formation of fine nanoparticles that are essential for effective catalysis. Under carefully optimized conditions, illustrated in Scheme 18, the catalyzed reaction yielded a broad spectrum of triaryl imidazole derivatives with remarkable efficiency, often achieving high to excellent yields in less than one hour. The intricate mechanism underlying the synthesis of 2,4,5-triaryl imidazoles is further elucidated in Scheme 19, showcasing the three-component reaction of aldehydes, benzene, and ammonium acetate, all catalyzed by the Fe_3_O_4_@SiO_2_–ABMA–MnCl_2_ nanocomposite under specific conditions. This methodology not only demonstrates the effectiveness of the nanocatalyst in facilitating complex chemical transformations but also emphasizes its significant potential for advancing sustainable practices in synthetic chemistry. The Fe_3_O_4_@SiO_2_–ABMA–MnCl_2_ nanocatalyst demonstrated impressive stability in recycling tests, maintaining its catalytic efficiency with only a slight decline after 8 uses. Analyses using VSM, TGA, and ICP-OES confirmed that the Fe_3_O_4_@SiO_2_–ABMA–MnCl_2_ catalyst exhibits high stability, maintaining its magnetic properties and structural integrity even after eight consecutive uses. This exceptional stability highlights the catalyst's reliability for repeated applications in sustainable chemical reactions.

**Scheme 20:**
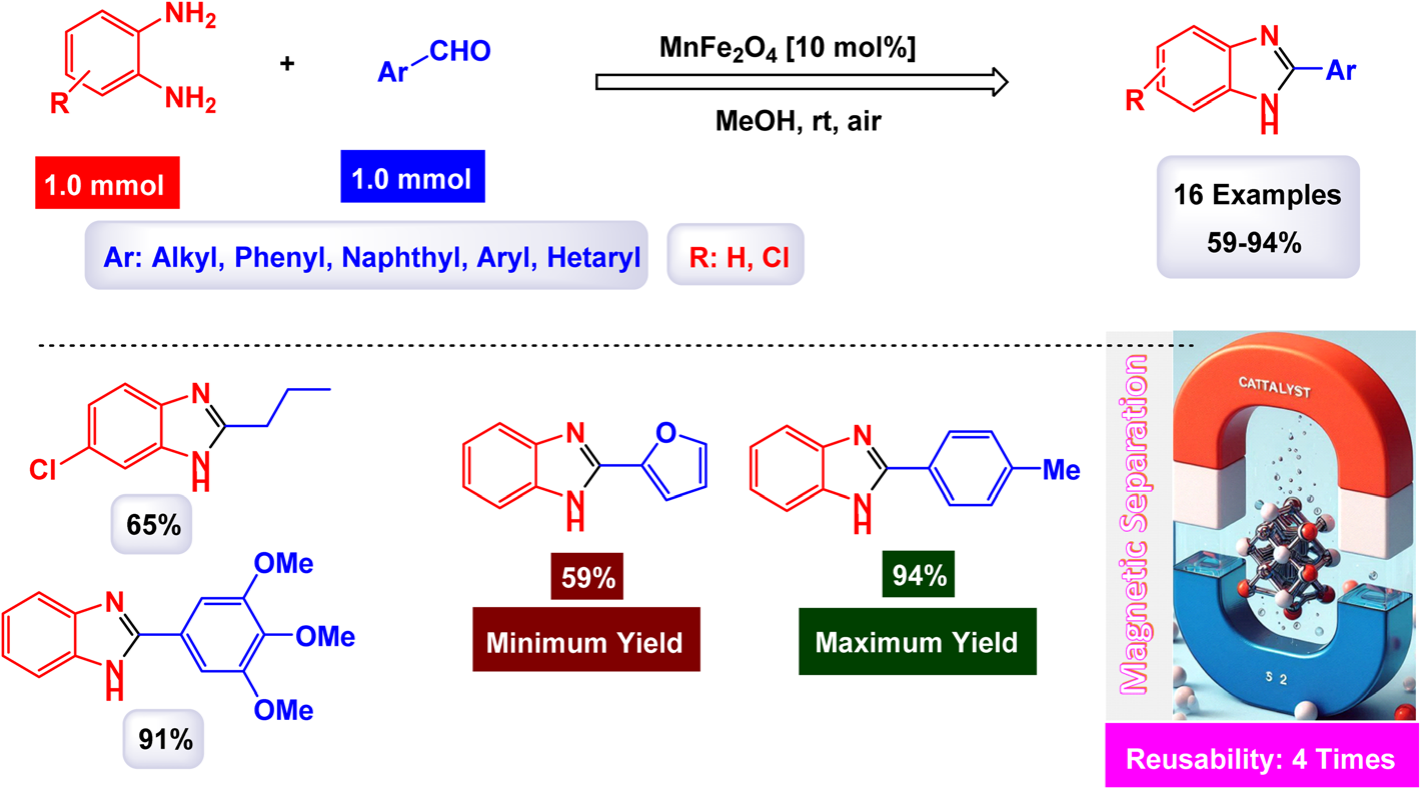
Synthesis of benzimidazoles [catalysis by MnFe_2_O_4_ nanocomposite].

**Scheme 21:**
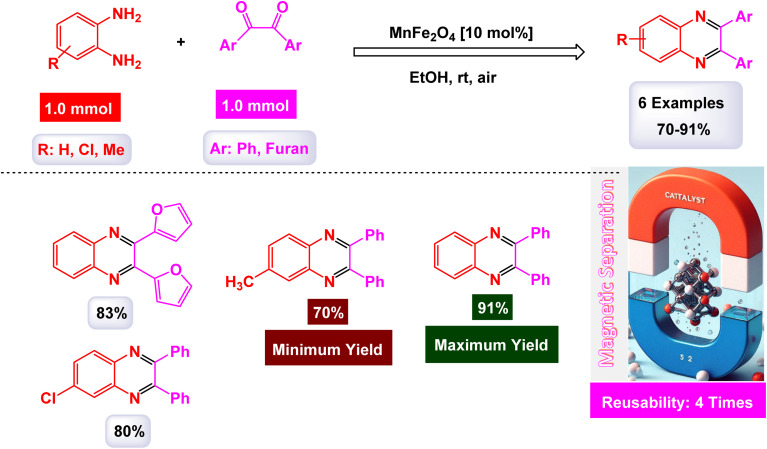
Synthesis of quinoxalines [catalysis by MnFe_2_O_4_ nanocomposite].

**Scheme 22:**
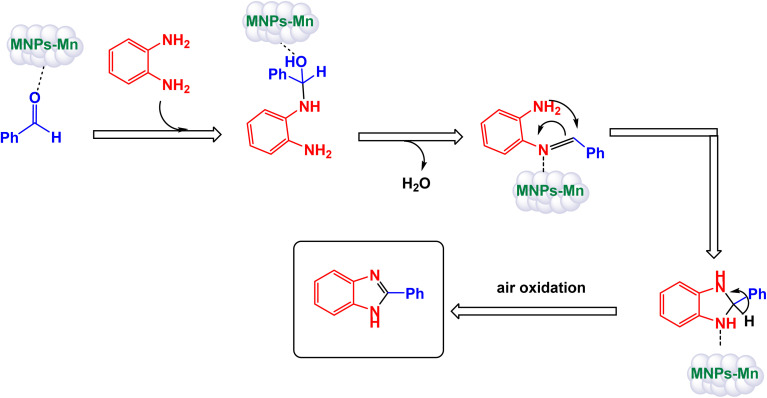
Suggested mechanism for synthesis of benzimidazoles [catalysis by MnFe_2_O_4_ nanocomposite].

Brahmachari and his research team have made a significant breakthrough in the synthesis of biologically important 2-substituted benzimidazoles and quinoxalines (Schemes 20 and 21).^146^ Their innovative protocol employs magnetically separable manganese ferrite (MnFe_2_O_4_) nanopowder, functioning as a highly effective and reusable heterogeneous catalyst. This allows reactions to take place effortlessly at room temperature and under aerobic conditions, enhancing convenience and efficiency. The characterization of the catalyst is impressive, revealing an average particle size of 50 nm *via* XRD, with TEM confirming nanocrystal sizes ranging from 50 to 100 nm. This cutting-edge method efficiently accommodates a wide variety of aromatic aldehydes—featuring both electron-donating and electron-withdrawing groups such as F, Cl, Br, CN, OH, OMe, tri-OMe, and Me—yielding striking results. The synthesis of 2-substituted benzimidazoles achieves yields between 59% and an outstanding 94%, all within a practical timeframe of just 4 to 20 hours at room temperature. The authors present a compelling mechanism for the simplification of forming these valuable compounds, showcasing MnFe_2_O_4_ dual role as both a Lewis acid and oxidative agent (Scheme 22). Importantly, recycling tests demonstrate that the MnFe_2_O_4_ nanopowder can be reused effectively for multiple reactions without a notable decrease in catalytic performance. This highlights the catalyst's exceptional sustainability and efficiency, marking a remarkable advance in facilitating this vital synthetic transformation.

#### 2.2.2. Synthesis of triazoles

Triazole derivatives are an important class of heterocyclic compounds with significant biological and pharmaceutical properties.^147^ The triazole ring, consisting of a five-membered structure with three nitrogen atoms, exhibits strong hydrogen bonding and electron-donating characteristics, making it highly versatile in drug design.^148,149^ Triazole-based compounds demonstrate a broad spectrum of biological activities, including antifungal, antibacterial, antiviral, anti-inflammatory, and anticancer effects.^150^ Notably, triazole derivatives like fluconazole and itraconazole are widely used antifungal agents that inhibit ergosterol biosynthesis, essential for fungal cell membrane integrity.^151,152^ Additionally, triazoles are found in antiviral drugs, including potential treatments for HIV and hepatitis C.^153^ Their strong metabolic stability, bioavailability, and ability to interact with biological targets make them valuable in pharmaceutical development, driving research into novel therapeutic applications.

**Scheme 23:**
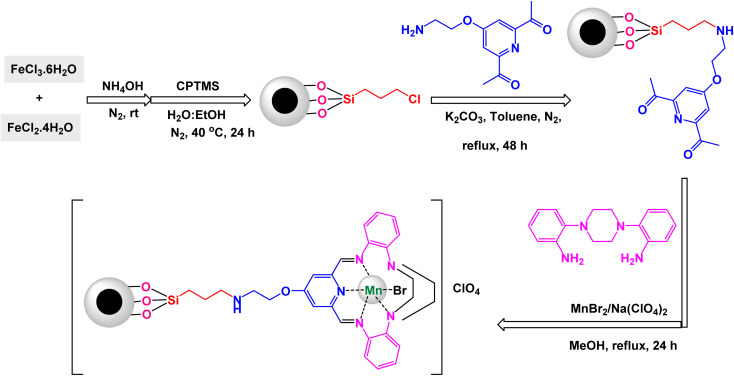
General method for construction of [Fe_3_O_4_@PAM–Schiff-base–Mn][ClO_4_] nanocomposite.

**Scheme 24:**
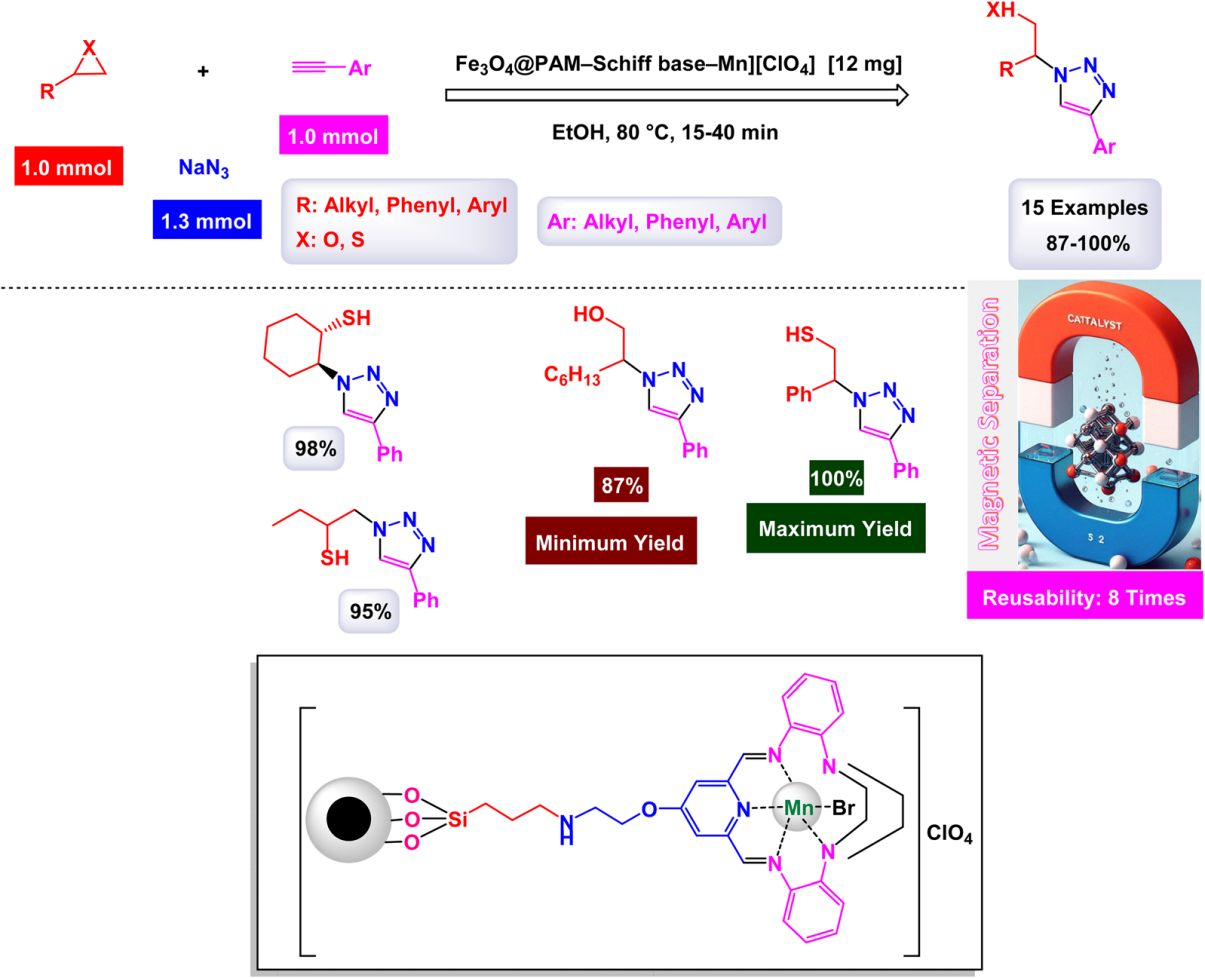
Synthesis of 1,2,3-substituted triazoles [catalysis by [Fe_3_O_4_@PAM–Schiff-base–Mn][ClO_4_] nanocomposite].

**Scheme 25:**
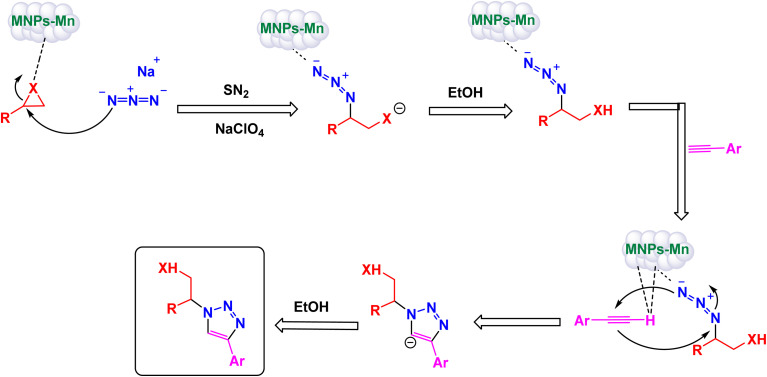
Suggested mechanism for synthesis of 1,2,3-substituted triazoles [catalysis by [Fe_3_O_4_@PAM–Schiff-base–Mn][ClO_4_] nanocomposite].

To prepare 1,2,3-substituted triazoles, Riadi and his research team have expertly designed a remarkable symmetrical 15-membered macrocyclic Schiff base complex of manganese nanomaterial, referred to as [Fe_3_O_4_@PAM–Schiff-base–Mn][ClO_4_]. This cutting-edge catalyst acts as a magnetically recoverable agent, significantly enhancing a one-pot, three-component reaction that elegantly combines styrene episulfides or styrene epoxides with various alkynes and sodium azide.^154^ The synthesis of the [Fe_3_O_4_@PAM–Schiff-base–Mn][ClO_4_] catalyst involves a meticulous reaction between 2,6-diacetylpyridine-functionalized magnetite nanoparticles (Fe_3_O_4_ MNPs) and 2,2-(piperazine-1,4-diyl)dianiline in the presence of manganese(ii) bromide, resulting in a sophisticated composite material (Scheme 23). TEM analysis has confirmed the successful creation of this manganese catalyst, revealing an average particle size ranging from 21 to 28 nanometers, which is crucial for achieving high catalytic activity. The catalyst also boasts impressive magnetic properties and remarkable thermal stability, as demonstrated by VSM and TGA. Under optimized and standardized reaction conditions, a diverse array of 2,3-triazoles was synthesized with striking efficiency. The process involves the reaction of various substituted epoxides and thiiranes with both aliphatic and aromatic terminal alkynes, in conjunction with sodium azide, resulting in high product yields while successfully circumventing any undesirable by-products (Scheme 24). The authors provide a detailed and insightful mechanism for the catalytic synthesis of 1,4-disubstituted-1,2,3-triazoles. This intricate mechanism outlines the Huisgen 1,3-dipolar cycloaddition reaction, which occurs between the activated substituted epoxide and thiirane, with alkynes and sodium azide, all skillfully catalyzed by the innovative [Fe_3_O_4_@PAM–Schiff base–Mn][ClO_4_] complex (Scheme 25). The M [Fe_3_O_4_@PAM–Schiff-base–Mn][ClO_4_] nanocatalyst demonstrated impressive stability in recycling tests, maintaining its catalytic efficiency with only a slight decline after 8 uses.

#### 2.2.3. Synthesis of pyrimidines

Pyrimidine derivatives are an essential class of heterocyclic compounds with significant biological and pharmaceutical properties.^155^ The pyrimidine ring, a six-membered structure with two nitrogen atoms, is a key component of nucleotides such as cytosine, thymine, and uracil, which are fundamental to DNA and RNA.^156,157^ Due to their structural similarity to natural nucleobases, pyrimidine derivatives exhibit diverse biological activities, including antiviral, antibacterial, antifungal, anti-inflammatory, and anticancer effects.^158,159^ They play a crucial role in chemotherapy, with drugs like 5-fluorouracil and cytarabine acting as potent antitumor agents by inhibiting DNA synthesis and cell proliferation. Additionally, pyrimidine-based compounds are used in antiviral medications, such as zidovudine for HIV treatment, and in cardiovascular drugs like minoxidil.^12,160^ Their broad-spectrum pharmacological activities and ability to target critical biological pathways make pyrimidine derivatives a vital area of research in drug discovery and medicinal chemistry.

**Scheme 26:**
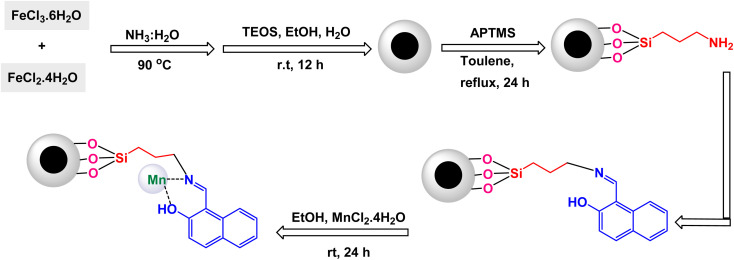
General method for construction of Fe_3_O_4_@SiO_2_@Mn-complex.

**Scheme 27:**
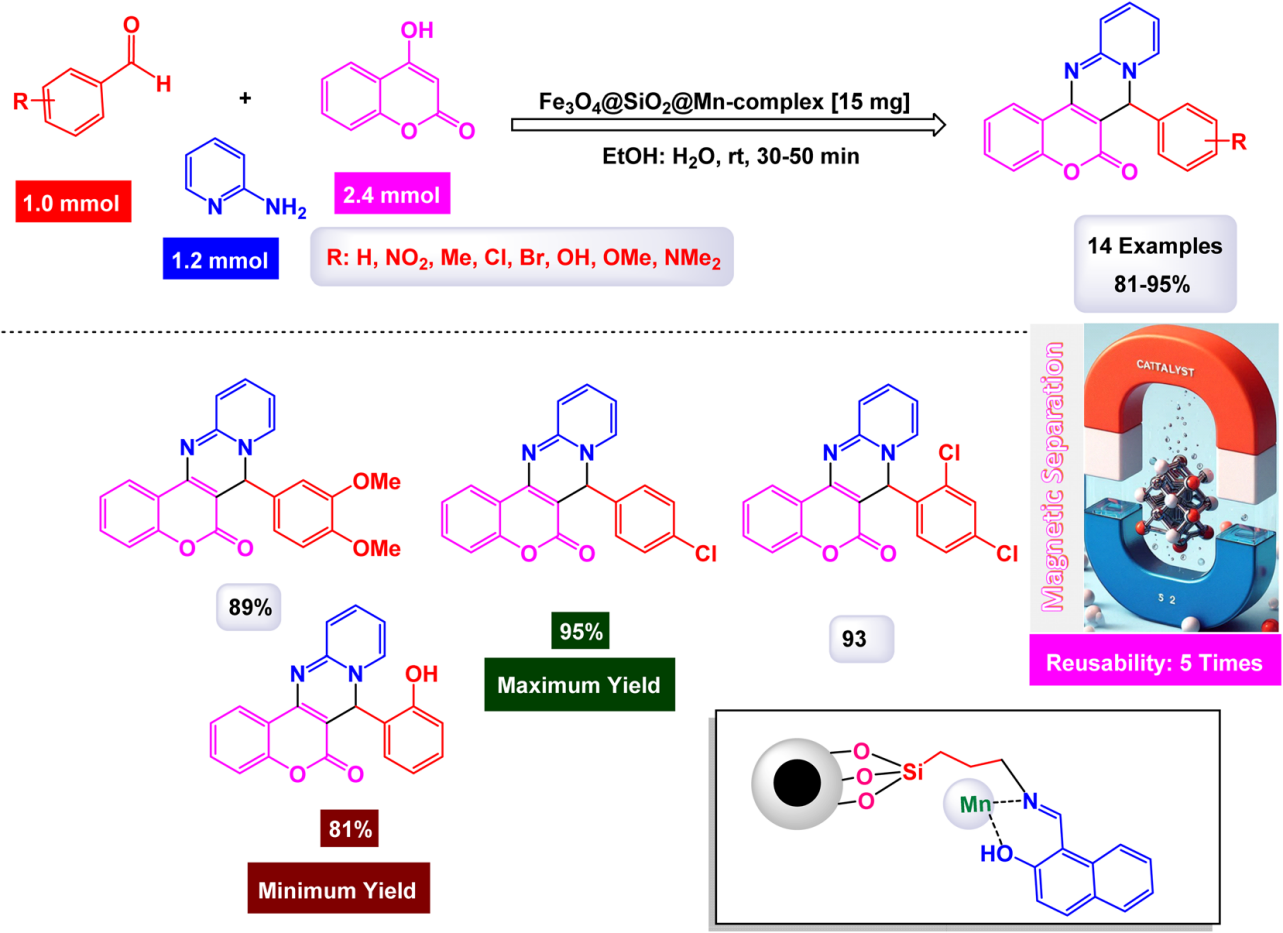
Synthesis of 7-aryl[4,3-*d*]pyrido[1,2-*a*]pyrimidin-6(7*H*)-ones [catalysis by Fe_3_O_4_@SiO_2_@Mn nanocomposite].

**Scheme 28:**
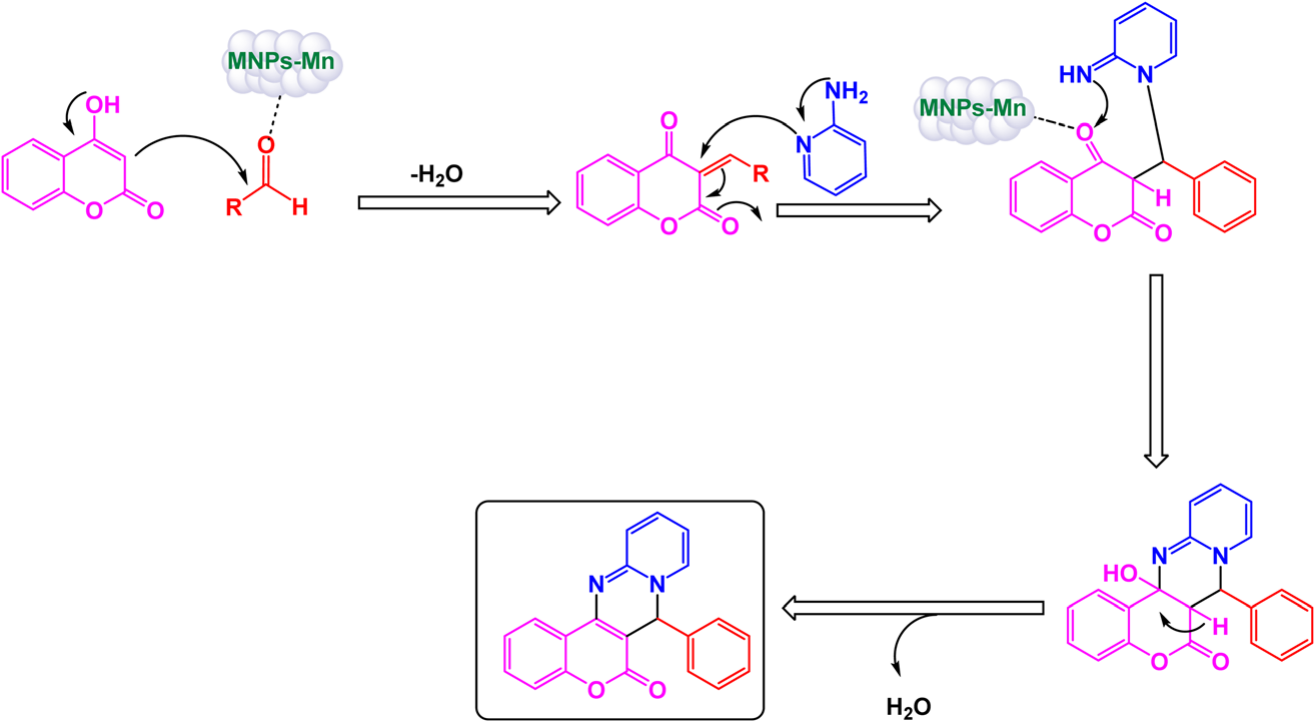
Suggested mechanism for synthesis of 7-aryl[4,3-*d*]pyrido[1,2-*a*]pyrimidin-6(7*H*)-ones [catalysis by Fe_3_O_4_@SiO_2_@Mn nanocomposite].

To prepare pyrimidine derivatives, Bodaghifard and his research team have successfully engineered a sophisticated hybrid magnetic nanocatalyst, identified as Fe_3_O_4_@SiO_2_@Mn-complex.^161^ This innovative catalyst integrates an immobilized Schiff base–Mn complex, enabling it to efficiently catalyze the synthesis of biologically active derivatives known as 7-aryl[4,3-*d*] pyrido[1,2-*a*]pyrimidin-6(7*H*)-one in an aqueous medium. The construction of this nanocatalyst employed a meticulous layer-by-layer assembly technique, which is beautifully illustrated in Scheme 26. The structural characteristics of the nanocatalyst were thoroughly examined through a range of analytical methods. XRD analysis revealed the crystallinity of the magnetic nanoparticles, showcasing a remarkably uniform crystallite size of approximately 15 nanometers, as indicated by the distinct peak observed at 2*θ* = 35.68° (311) in the diffraction pattern. SEM further complemented this characterization by providing visual confirmation of the nanoparticle morphology. As demonstrated in Scheme 27, the catalytic reaction proceeded with exceptional efficiency, leading to the formation of the desired chromeno[4,3-*d*]pyrido[1,2-*a*]pyrimidin-6-one derivatives. This process was characterized by high yield results and notably short reaction times, all while avoiding the formation of any undesirable side products, which is often a challenge in synthetic chemistry. The underlying mechanism of this transformation, elaborated in Scheme 28, begins with the activation of the carbonyl group of the aldehyde by a Lewis acid, which prepares the substrate for further reaction. This activation results in the formation of a key intermediate through a condensation reaction with 4-hydroxycoumarin. Following this step, a nucleophilic attack initiated by 2-aminopyridine occurs, which leads to a tautomerization process involving keto–enol forms, yielding another intermediate. The culmination of this chemical transformation is achieved through intramolecular ring closure of intermediate, ultimately finalizing the reaction and producing the target compound with high efficiency and selectivity. The Fe_3_O_4_@SiO_2_@Mn nanocatalyst demonstrated impressive stability in recycling tests, maintaining its catalytic efficiency with only a slight decline after 5 uses.

#### 2.2.4. Synthesis of dihydropyridines

Dihydropyridine derivatives are a crucial class of heterocyclic compounds known for their significant biological and pharmaceutical properties, particularly in cardiovascular medicine.^162^ Structurally, dihydropyridines (DHPs) are hydrogenated derivatives of pyridine, featuring a six-membered ring with one nitrogen atom.^163^ They are best known for their role as calcium channel blockers (CCBs), which selectively inhibit L-type calcium channels, leading to vasodilation and reduced blood pressure. This makes DHP derivatives, such as nifedipine, amlodipine, and felodipine, essential in treating hypertension, angina, and other cardiovascular disorders.^164,165^ Beyond their cardiovascular applications, DHPs have demonstrated neuroprotective, anti-inflammatory, and anticancer activities, with ongoing research exploring their potential in neurodegenerative diseases like Alzheimer's.^166,167^ Their favorable pharmacokinetic properties, including good bioavailability and metabolic stability, make them highly valuable in drug development, ensuring their continued importance in modern therapeutics.

**Scheme 29:**
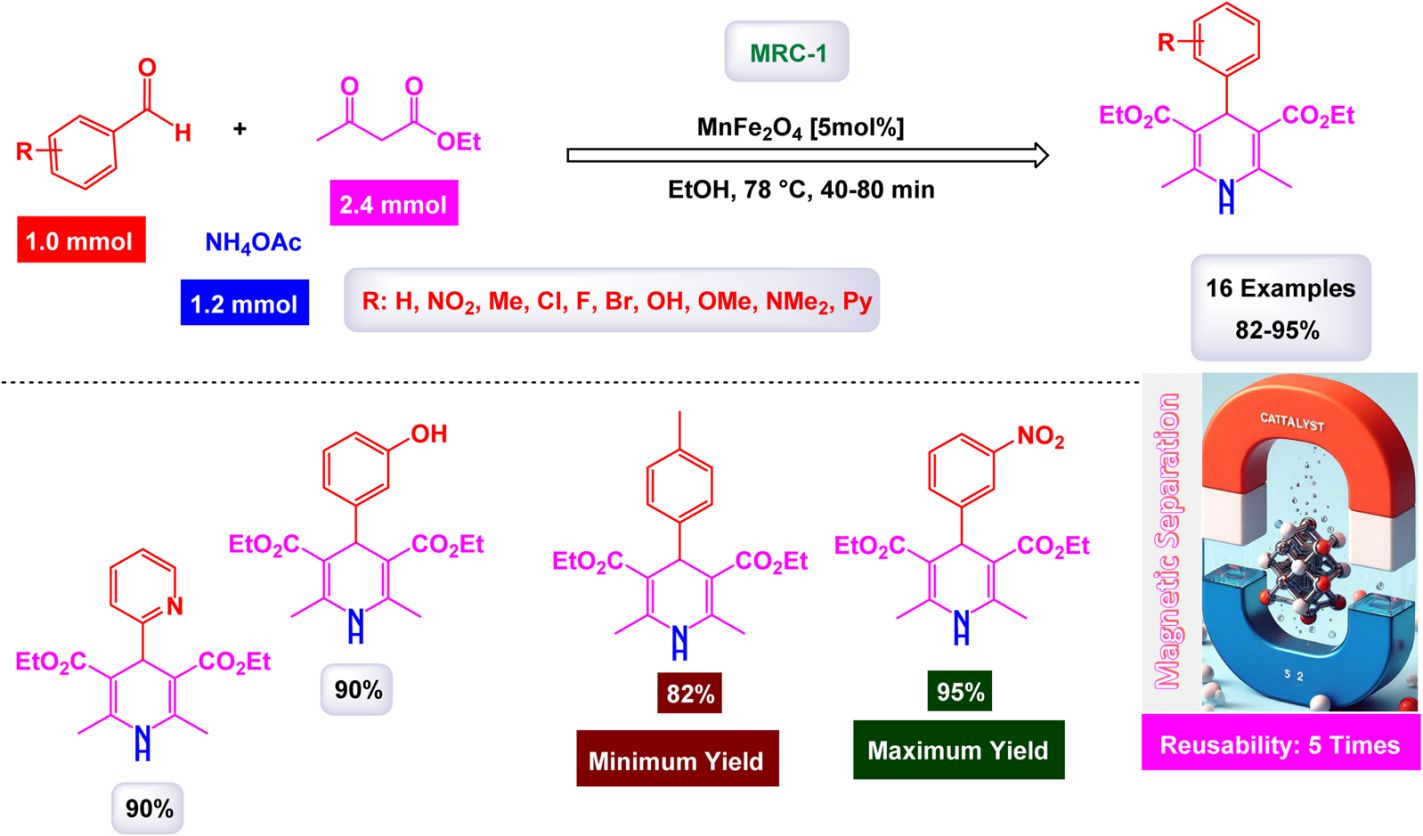
Synthesis of 1,4-dihydropyridines [catalysis by MnFe_2_O_4_ nanocomposite].

Naeimi and his research team have introduced an innovative and highly efficient one-pot synthesis method designed specifically for the preparation of various 1,4-dihydropyridines.^168^ This groundbreaking approach involves the meticulous condensation of carefully selected aldehyde derivatives, ethyl acetoacetate, and ammonium acetate, all performed in the presence of superparamagnetic manganese ferrite nanoparticles. The reaction is conducted at a precise controlled temperature of 80 °C, which is crucial for optimizing product yields. The manganese ferrite nanoparticles used in this synthesis possess a notably small particle size of approximately 33 nm. This measurement was obtained through an in-depth analysis of the line broadening at half the maximum intensity (full width at half maximum, FWHM) at a specific diffraction angle of 2*θ* = 35.31, utilizing the Debye–Scherrer equation, defined as *D* = 0.9*λ*/*β* cos *θ*. Additionally, the remarkable magnetic properties of these MnFe_2_O_4_ nanoparticles were corroborated using the VSM technique, which confirmed their superparamagnetic behavior, making them ideal for separation and recovery after the reaction. A striking observation from this study is the stark difference in yields based on the electronic characteristics of the aldehyde derivatives used. Specifically, benzaldehyde derivatives containing electron-withdrawing groups, such as 3-nitrobenzaldehyde and 4-chlorobenzaldehyde, were found to produce exceptionally high quantities of the desired products. In contrast, when aldehyde derivatives with electron-donating groups were utilized, the reaction yielded significantly lower amounts of product (Scheme 29). This finding highlights the critical role of electronic effects in influencing the outcomes of the condensation reaction. Overall, this synthesis method stands out as a promising advancement in the effective preparation of 1,4-dihydropyridines, showcasing the potential for further exploration within this area of organic chemistry. The MnFe_2_O_4_ nanocatalyst demonstrated impressive stability in recycling tests, maintaining its catalytic efficiency with only a slight decline after 5 uses.

#### 2.2.5. Synthesis of imidazopyridines

Imidazopyridine derivatives are an important class of fused heterocyclic compounds that exhibit a wide range of biological and pharmaceutical properties.^169^ Combining the structural features of imidazole and pyridine, these compounds possess enhanced electron-donating and hydrogen-bonding capabilities, making them highly effective in drug design.^170^ Imidazopyridine derivatives are known for their diverse pharmacological activities, including anti-inflammatory, antimicrobial, antiviral, anticancer, and neuroactive effects.^171^ Notably, they are widely used as central nervous system (CNS) agents, with drugs like zolpidem acting as non-benzodiazepine hypnotics for treating insomnia by modulating GABA-A receptors.^172^ Additionally, these compounds have shown promise in targeting kinases and other enzymes involved in cancer and infectious diseases.^173^ Their strong bioavailability, metabolic stability, and ability to interact with various biological targets make imidazopyridine derivatives valuable candidates for developing novel therapeutics.

**Scheme 30:**
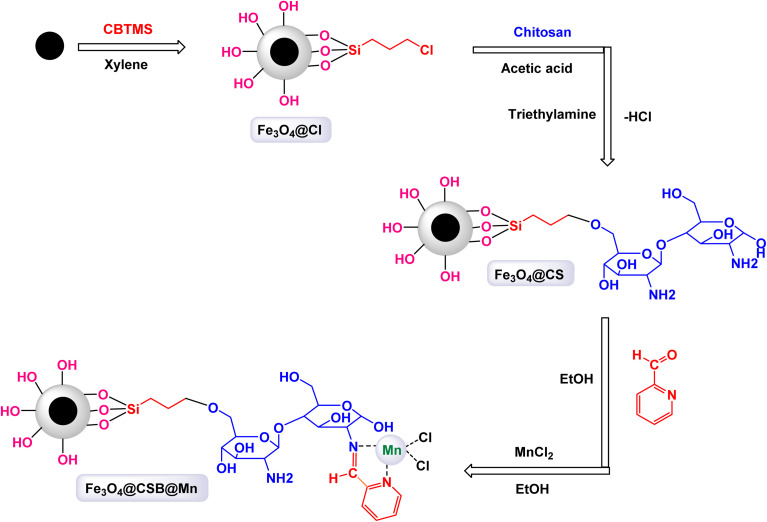
General method for construction of Fe_3_O_4_@CSBMn nanocomposite.

**Scheme 31:**
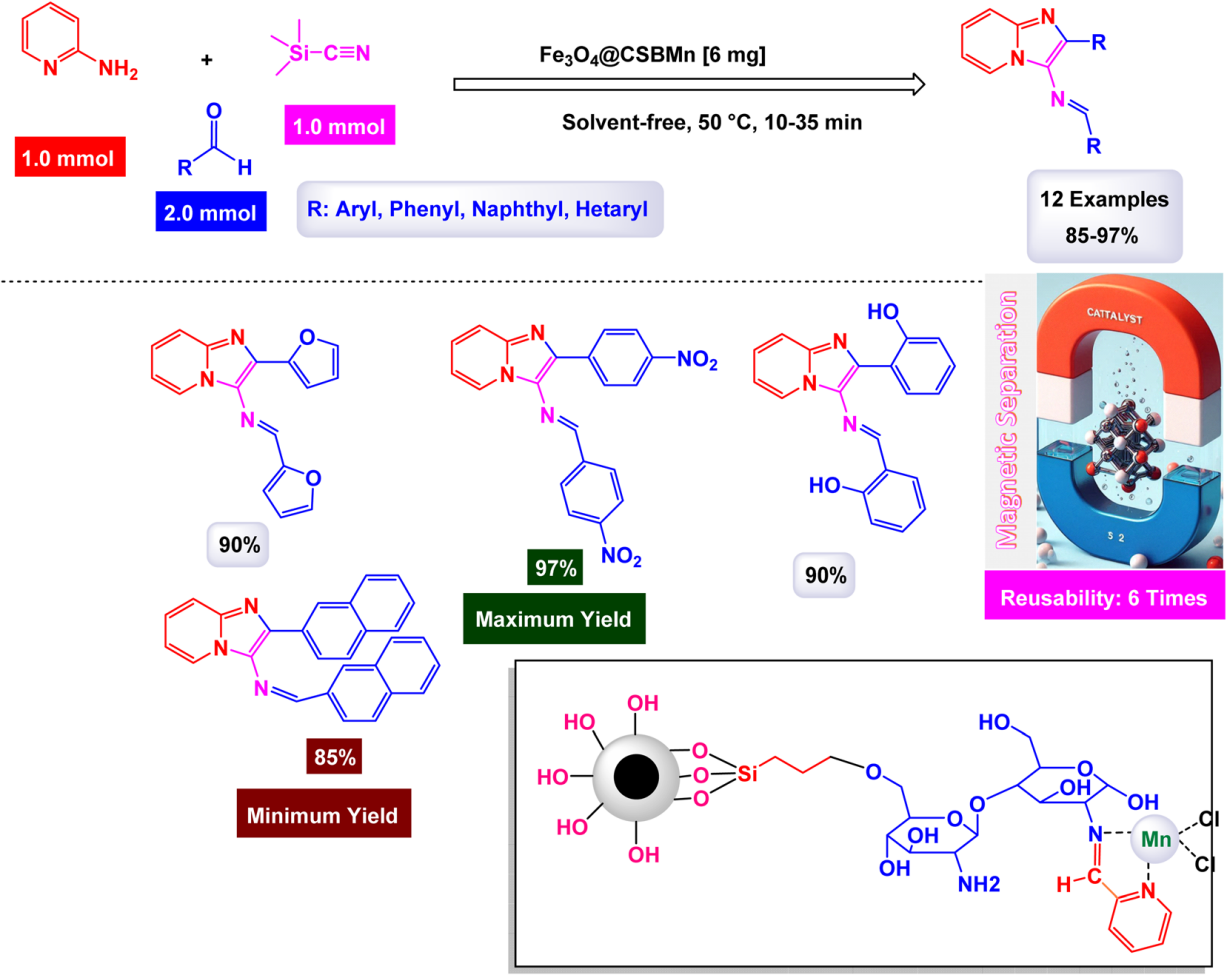
Synthesis of imidazo[1,2-*a*]pyridines [catalysis by Fe_3_O_4_@CSBMn nanocomposite].

**Scheme 32:**
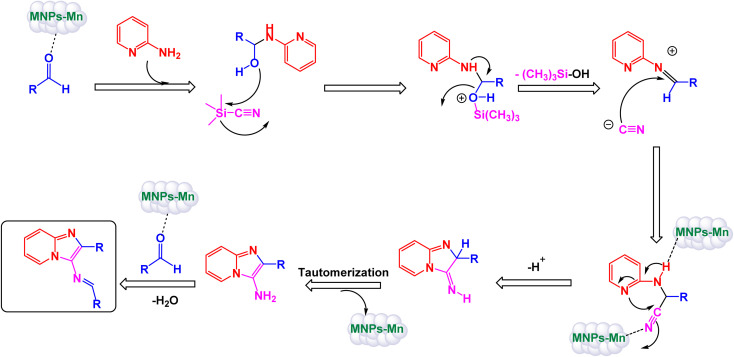
Suggested mechanism for synthesis of imidazo[1,2-*a*]pyridines [catalysis by Fe_3_O_4_@CSBMn nanocomposite].

In a paper published by Jamshid Rakhtshah *et al.*,^174^ magnetic nanoparticles (Fe_3_O_4_) were prepared and coated with 3-chloropropyl(trimethoxy)silane followed by a chitosan layer to prepare the corresponding chitosan-coated metal nanoparticles. After that, 2-formylpyridine was added to form the Schiff base. Finally, manganese acetate was added to obtain a manganese Schiff base complex immobilized on metal nanoparticles (Fe_3_O_4_@CSBMn) as shown in Scheme 30. FT-IR analysis shows the presence of absorption bands at 1620 cm−1 due to C=N present in Schiff base in addition to shifting to a frequency after the addition of manganese acetate indicating the binding of N to manganese ions. The pattern of Fe_3_O_4_ in the XRD spectrum is consistent with the standard sample and the spectrum did not change after the addition of organic layers indicating its stability. SEM analysis shows the spherical shape and nano-sized size of the synthesized catalyst, with the presence of C, N, O, Cl, Si, Fe, and Mn elements as shown in EDS analysis, indicating the purity of the synthesized catalyst. Through VSM analysis, a decrease in the magnetic saturation value was observed due to the layers fixed on the magnetic nanoparticle Fe_3_O_4_. After the prepared catalyst was characterized and its functional groups and properties were confirmed, it was tested in the synthesis of 3-iminoaryl-imidazo[1,2-*a*]pyridine through a three-component reaction in one pot. The reaction was carried out in the presence of benzaldehyde, TMSCN, and 2-aminopyridine in the presence of (6 mg) of (Fe_3_O_4_@CSBMn) as a catalyst in a solvent-free medium at 50 °C as shown in Scheme 31. After testing a different set of aldehydes, it was found that aromatic aldehydes with electron-donating groups give weaker yields than those with electron-withdrawing groups. The Fe_3_O_4_@CSBMn nanocatalyst demonstrated impressive stability in recycling tests, maintaining its catalytic efficiency with only a slight decline after 6 uses. A mechanism for the formation of IAIP derivatives is outlined in Scheme 32, consisting of three main steps. In the first step, the activated carbonyl group of the aldehyde (II) reacts with a manganese complex immobilized on Fe_3_O_4_ (I) and 2-aminopyridine (III). This reaction leads to the formation of intermediate (IV), with the amine (III) acting as a more potent nucleophile than TMSCN (V). In the second step, TMSCN (V) interacts with intermediate (IV) to generate a new intermediate (VI) and release trimethylsilyl hydroxide, forming intermediate (VII). The final step features a nucleophilic addition of a cyanide anion to intermediate (VII), resulting in intermediate (VIII). This undergoes cyclization to become intermediate (IX) and subsequently tautomerizes to form intermediate (X). The process concludes with the addition of aldehyde (II) to intermediate (X), leading to the formation of the desired 3-iminoaryl-imidazo[1,2-*a*]pyridines (XI) through imine bond formation.

### 2.3. Coupling reactions

Catalysis plays a crucial role in coupling reactions by enabling the formation of complex molecules through efficient bond formation.^10,175^ Many coupling reactions, such as Suzuki, Heck, and Sonogashira reactions, rely on catalysts–often transition metals like palladium, nickel, or copper—to facilitate the process.^176^ These catalysts lower the activation energy required for bond formation, making the reaction more efficient and selective.^177,178^ Without catalysts, many coupling reactions would proceed too slowly or require extreme conditions, making them impractical for industrial and pharmaceutical applications. In addition to improving reaction efficiency, catalysis enhances selectivity in coupling reactions, ensuring that the desired product is obtained with minimal side reactions or byproducts.^179^ This is particularly important in the synthesis of pharmaceuticals, agrochemicals, and advanced materials, where purity and yield are critical. By fine-tuning the choice of catalyst, ligands, and reaction conditions, chemists can control reaction pathways and achieve high regio- and stereoselectivity.^180,181^ This level of precision is essential for creating complex molecular architectures that would otherwise be challenging or impossible to obtain. From an economic and environmental perspective, catalytic coupling reactions contribute to sustainability by reducing waste and energy consumption.^182,183^ Many traditional synthetic methods require stoichiometric amounts of reagents, leading to excess byproducts and high costs. In contrast, catalytic methods often operate under milder conditions and use smaller amounts of expensive or hazardous reagents, making them more environmentally friendly and cost-effective.^184^ Advances in catalyst design, including the development of recyclable and earth-abundant catalysts, continue to drive progress toward greener and more sustainable chemical processes.

Manganese-based magnetic catalysts are gaining significant attention in coupling reactions due to their unique combination of catalytic efficiency, cost-effectiveness, and ease of recovery. Manganese, being an earth-abundant and environmentally benign transition metal, serves as an excellent alternative to expensive noble metals like palladium or platinum. In coupling reactions, manganese catalysts facilitate the formation of carbon–carbon and carbon–heteroatom bonds, often under mild conditions, enhancing reaction efficiency and selectivity. Additionally, the magnetic properties of these catalysts allow for easy separation from reaction mixtures using an external magnetic field, eliminating the need for complex filtration or purification steps. This feature not only simplifies catalyst recovery but also enhances catalyst recyclability, making the process more sustainable. The use of magnetic manganese catalysts also contributes to greener and more economical chemical synthesis. Traditional coupling reactions often involve hazardous reagents and generate large amounts of waste, but manganese-based catalysts can operate under milder, more environmentally friendly conditions, reducing the need for toxic solvents or excessive reagent use. Their high reusability further minimizes waste and lowers production costs, making them attractive for large-scale industrial applications, including pharmaceutical and fine chemical manufacturing. As research continues to refine these catalysts, their role in sustainable catalysis and green chemistry will likely expand, offering an eco-friendly alternative to traditional catalytic systems.

#### 2.3.1. Synthesis of nitriles

Nitriles play a crucial role in medicinal and biological chemistry due to their versatile functionality and ability to serve as building blocks for various pharmacologically active compounds.^185^ Their presence in drug design enables the development of diverse therapeutic agents, including anti-cancer and anti-inflammatory medications.^186^ Moreover, nitriles can enhance the lipophilicity and metabolic stability of molecules, making them more effective in biological systems.^187^ The synthesis of nitriles is equally significant, as it provides chemists with accessible methods to create these valuable compounds efficiently.^188^ The development of reliable synthetic pathways facilitates the exploration of novel nitrile derivatives, ultimately advancing research in drug discovery and biochemistry.^189^

**Scheme 33:**
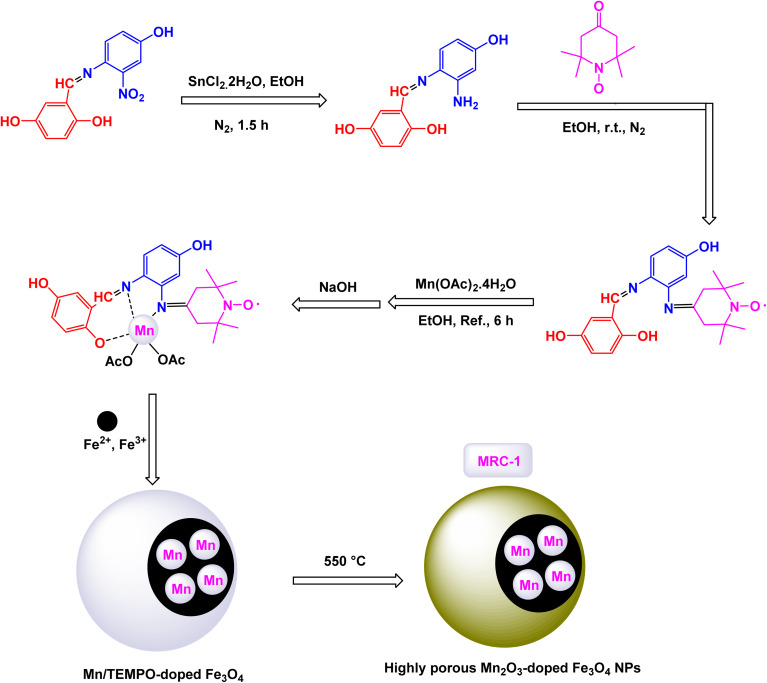
General method to construct Mn_2_O_3_-doped Fe_3_O_4_ NPs nanocomposite [MRC-1].

**Scheme 34:**
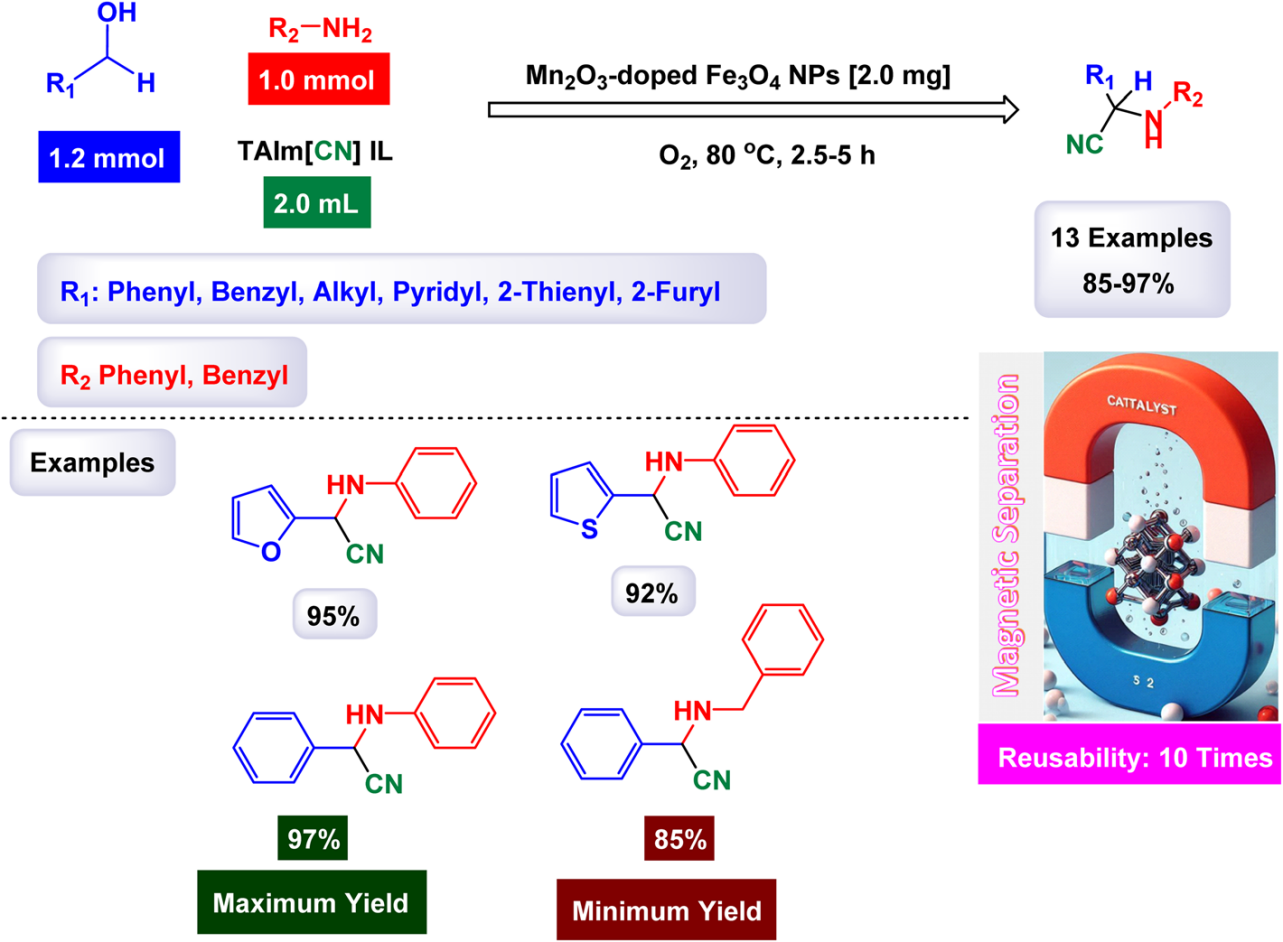
Synthesis of alpha-aminonitriles from alcohols and amines [catalysis by Mn_2_O_3_-doped Fe_3_O_4_ NPs nanocomposite].

**Scheme 35:**
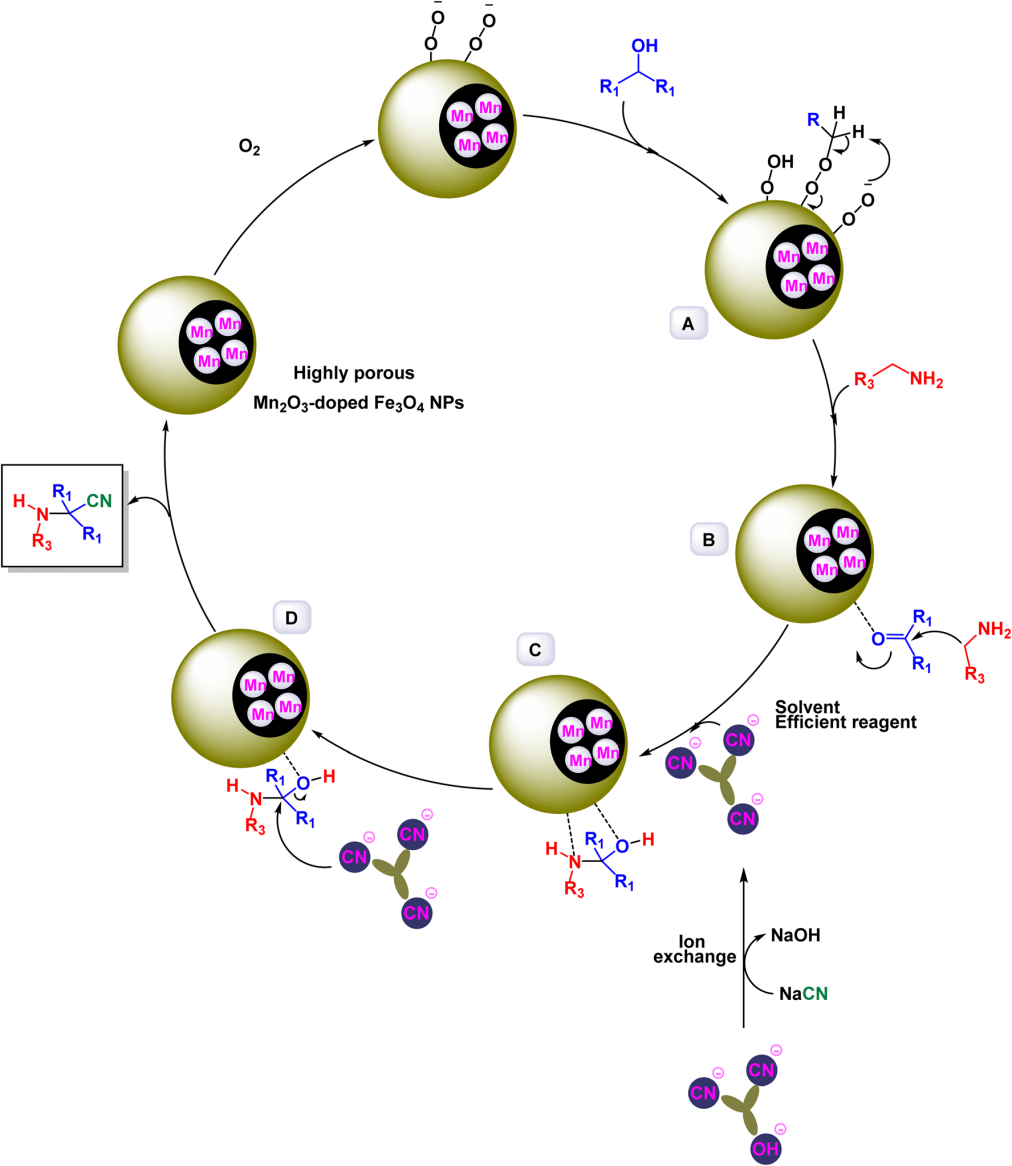
Synthetic pathway for synthesis of alpha-aminonitriles from alcohols and amines [catalysis by Mn_2_O_3_-doped Fe_3_O_4_ NPs nanocomposite].

The research team of Milad Kazemnejadi has developed a new magnetic nanocatalyst of Mn_2_O_3_-doped Fe_3_O_4_ NPs for the preparation of alpha-aminonitriles *via* Strecker synthesis from primary and secondary alcohols under mild conditions with TAIm[CN] as solvent and reagent.^190^ First, Mn/TEMPO-Salen was prepared as a complex, reducing agent and template for the synthesis of Mn/TEMPO-doped Fe_3_O_4_ magnetic particles, and to increase the porosity of the composite, the thermal decomposition process was carried out as shown in the Scheme 33. The highly porous nanoparticles were characterized by FTIR, BET, TGA, VSM, ICP, EDX, TEM, XRD, UV-Vis, and XPS analyses. FTIR analysis of Mn_2_O_3_-doped Fe_3_O_4_ NPs confirmed the presence of vibrational bands due to Mn–O and Fe–O–Mn, EDX and ICP analysis of Mn_2_O_3_-doped Fe_3_O_4_ NPs indicated the presence of 10.2 wt% and 10.34 wt% of manganese respectively, by zeta potential analysis the zeta potential value was −15 mV which indicates the stability of the nanocomposite and prevents its agglomeration. The catalytic activity of Mn_2_O_3_-doped Fe_3_O_4_ NPs in the preparation of α-aminonitriles from primary and secondary alcohols was evaluated in the presence of TAIm[CN]IL as an active reagent, a source of [CN], and an active solvent by the reaction of alcohol and amine, and 2.0 mg of Mn_2_O_3_-doped Fe_3_O_4_ NPs as a catalyst for the preparation of primary alcohols (Scheme 34). Then, the direct conversion of secondary alcohols was carried out under a volumetric flow rate of O_2_ of 20 mL min^−1^ at 80 °C. It gave yields of 85 to 97% and a time of 40 to 90 min. The Mn/TEMPO-doped Fe_3_O_4_ nanocatalyst, demonstrated impressive stability in recycling tests, maintaining its catalytic efficiency with only a slight decline after 10 uses. The synthetic pathway in Scheme 35 begins with the abstraction of a benzylic proton by the adsorbed –O–O– groups on the nanoparticles (NPs), yielding an aldehyde product. When an amine is present, it enables a nucleophilic attack on the aldehyde at the NP surface, resulting in intermediate C and then intermediate B. The ionic liquid TAIm[CN] acts as both a solvent and a reagent, facilitating the transfer of the cyanide ion ([CN]) to intermediate IV. This nucleophilic attack produces the desired α-aminonitrile, paralleling the role of [CN] ions in a bifunctional catalyst with [CN] counter anions. Moreover, the hydroxyl (OH) groups released from intermediate D are absorbed by TAIm[CN], allowing the catalyst to regenerate and re-enter the reaction cycle, enhancing overall efficiency.

#### 2.3.2. Synthesis of indoles

Indoles are biologically and pharmaceutically significant heterocyclic compounds that serve as key structural components in many natural and synthetic bioactive molecules.^191^ They are found in essential biomolecules like tryptophan, an amino acid crucial for protein synthesis and neurotransmitter production, as well as in hormones such as serotonin and melatonin, which regulate mood and sleep.^191^ In pharmaceuticals, indole derivatives exhibit a broad spectrum of therapeutic activities, including anticancer, antimicrobial, anti-inflammatory, and neuroprotective effects.^192^ Many clinically approved drugs, such as indomethacin (an anti-inflammatory agent) and vinblastine (a chemotherapeutic drug), are based on the indole scaffold. Additionally, indoles play a crucial role in plant growth regulators like auxins, further emphasizing their biological importance.^193^ Their versatile bioactivity makes them a valuable target in medicinal chemistry for developing novel therapeutics.

**Scheme 36:**
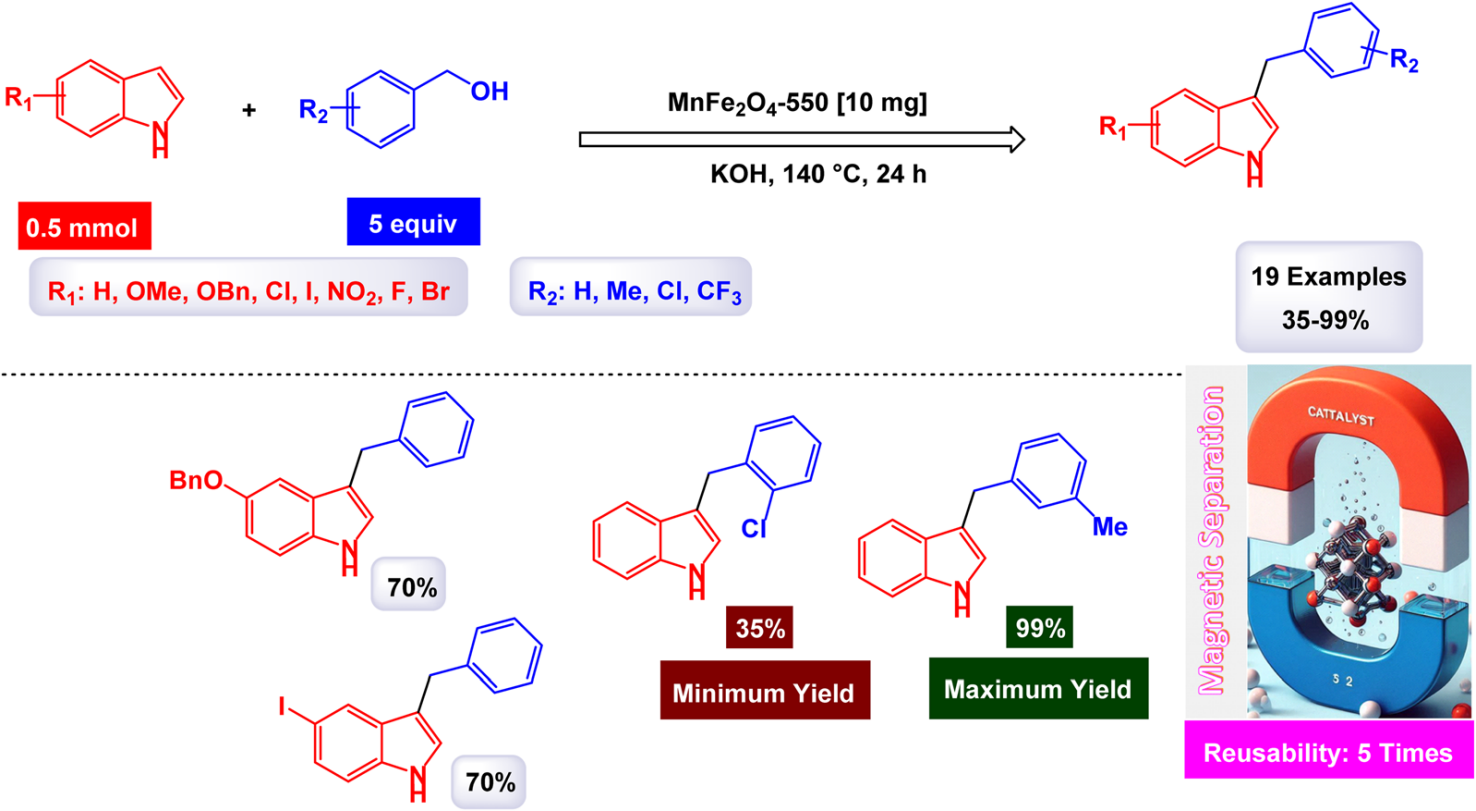
Synthesis of 3-benzylated indoles [catalysis by MnFe_2_O_4_ nanocomposite].

**Scheme 37:**
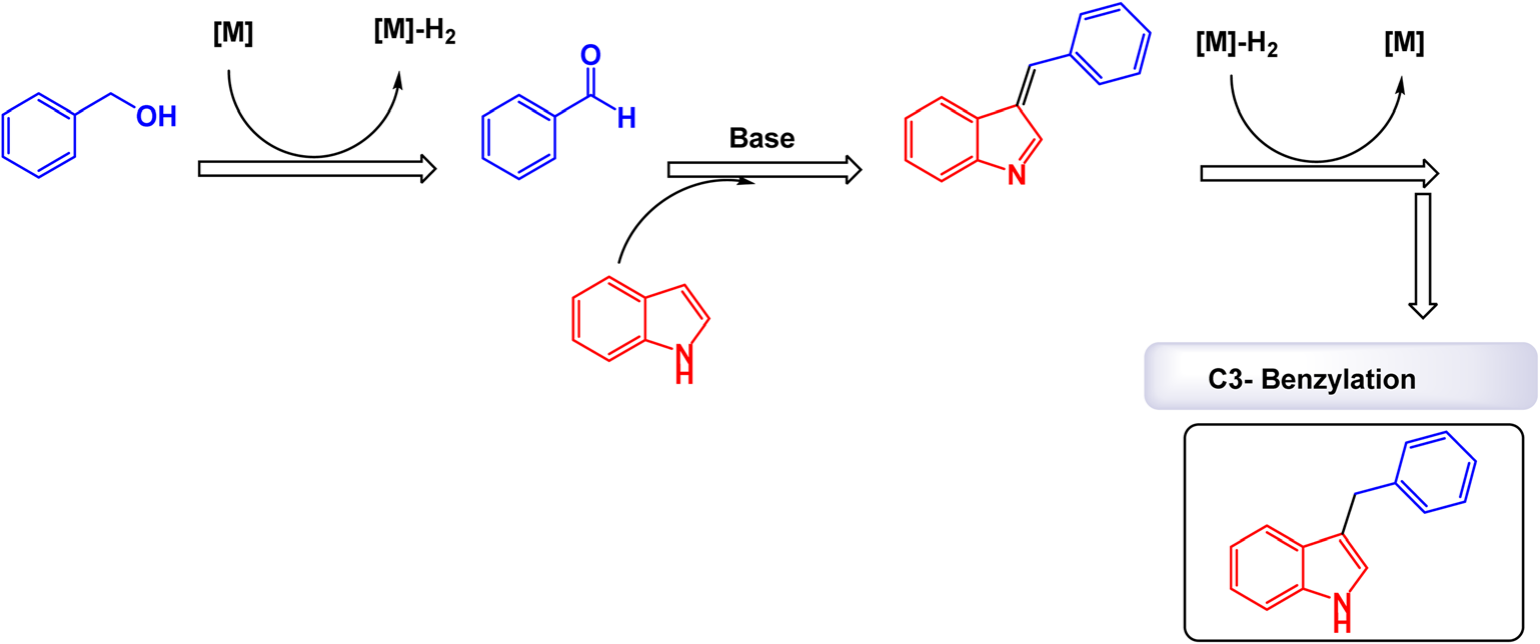
Suggested mechanism for synthesis of 3-benzylated indoles [catalysis by MnFe_2_O_4_ nanocomposite].

Tran and his research team have pioneered a groundbreaking one-pot reaction method to efficiently synthesize 3-benzylated indoles using MnFe_2_O_4_ nanoparticles as a heterogeneous catalyst.^194^ This cutting-edge process allows for the seamless coupling of benzyl alcohols with indoles, demonstrating particularly impressive results with electron-donating groups like 5-methoxyindole and 5-benzyloxyindole (Scheme 36). The methodology consistently yields products with outstanding isolated yields, making it both effective and reliable. Perhaps most notably, this innovative approach emphasizes sustainability through the incorporation of a recyclable oxide catalyst, which significantly enhances its eco-friendliness. By operating under solvent-free conditions and utilizing green, cost-effective alkylating reagents, this reaction method represents a remarkable advancement in environmentally conscious chemistry. The research also reveals that the average crystalline size of the MnFe_2_O_4_ nanoparticles increased from 4.4 nm to 6.5 nm, as confirmed by Debye–Scherrer's equation. This finding aligns perfectly with TEM analysis, ensuring that the catalyst's structural integrity is preserved throughout the reaction process. Overall, this method offers an exceptional and sustainable solution for synthesizing 3-benzylated indoles, showcasing a promising avenue for future research and industrial applications. The MnFe_2_O_4_ nanocatalyst, demonstrated impressive stability in recycling tests, maintaining its catalytic efficiency with only a slight decline after 5 uses. The authors present a compelling mechanism that clearly illustrates the straightforward synthesis of 3-benzylated indoles using MnFe_2_O_4_ nanoparticles as catalysts under specified experimental conditions. This innovative process is effectively depicted in Scheme 37, which highlights the crucial steps and interactions that define the reaction pathway. By providing a detailed visual representation, the authors enhance understanding of the underlying processes, showcasing the efficiency and elegance of this method in organic synthesis.

**Scheme 38:**
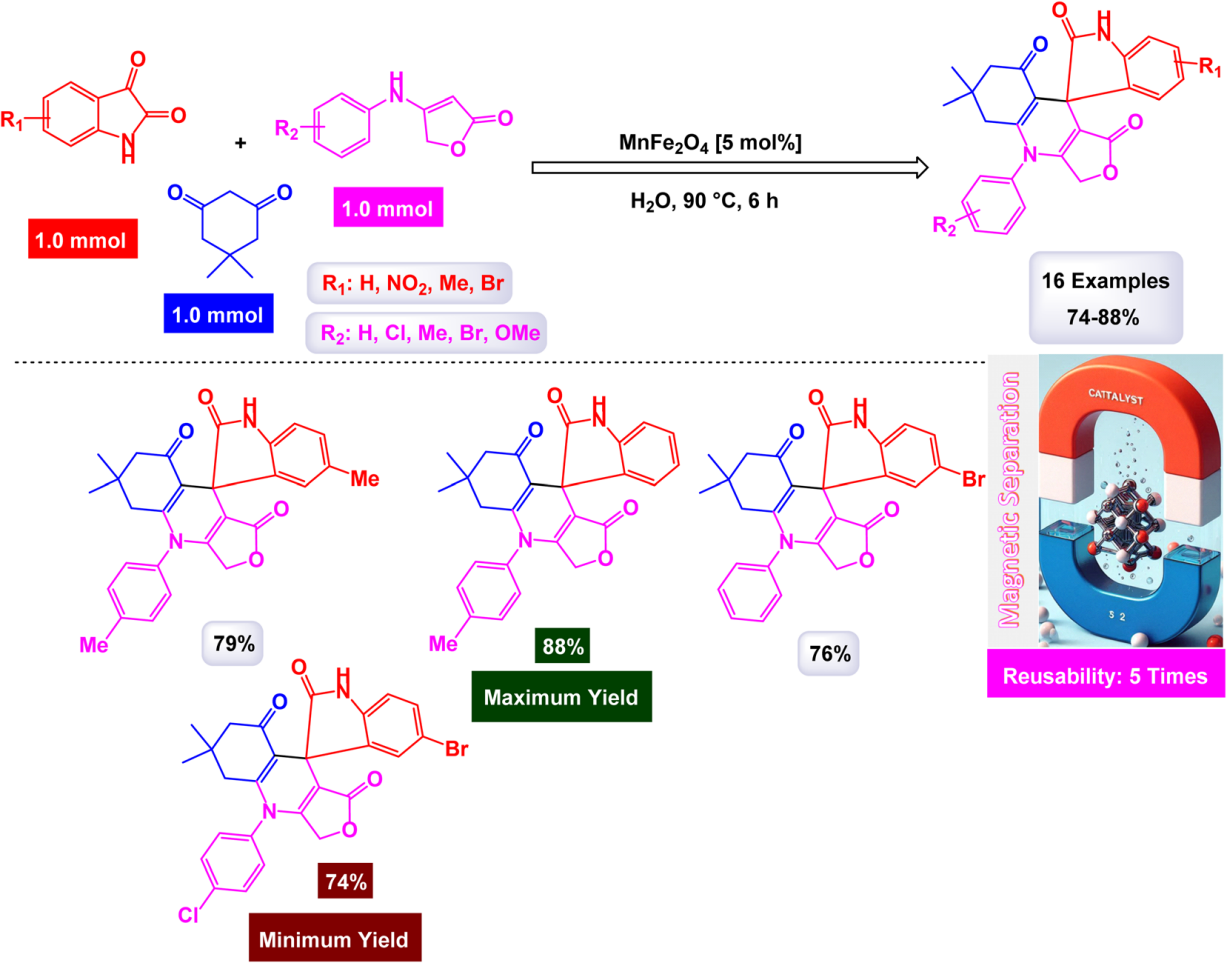
Synthesis of spirooxindole derivatives [catalysis by MnFe_2_O_4_ nanocomposite].

**Scheme 39:**
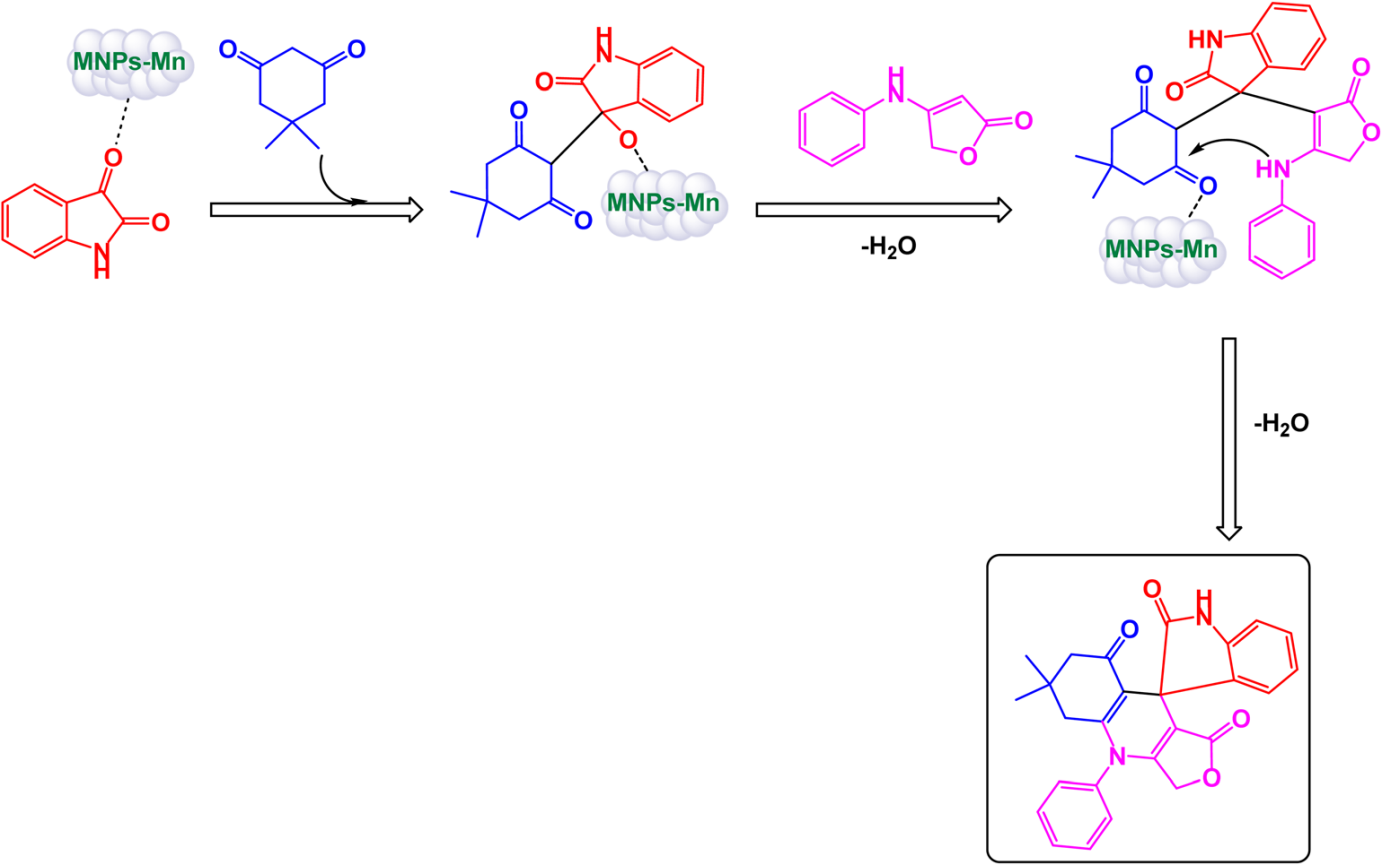
Suggested mechanism for synthesis of spirooxindole derivatives [catalysis by MnFe_2_O_4_ nanocomposite].

A novel and efficient method for synthesizing spirooxindole derivatives has been developed through a one-pot, three-component reaction involving isatins, dimedone, and anilinolactones (Scheme 38). Conducted by Naeimi and his team, this process utilizes manganese ferrite (MnFe_2_O_4_) nanoparticles as a highly effective and magnetically recoverable catalyst in water, which is considered a green solvent.^195^ The SEM images show that these nanoparticles have a mean diameter of approximately 33 nanometers and exhibit a nearly spherical shape. Their favorable magnetic and superparamagnetic properties are crucial for practical catalytic applications, as confirmed by VSM analysis. In the proposed mechanism (Scheme 39), the activated isatin reacts with dimedone under the influence of nano MnFe_2_O_4_ acting as a Lewis acid, leading to the formation of a reactive intermediate. This intermediate subsequently reacts with anilinolactone, resulting in the formation of spirooxindoles through a cyclization process, all driven by the catalytic action of the nanoparticles. This method not only streamlines the synthesis of spirooxindole derivatives but also enhances atom economy, marking a significant advancement in the field of organic synthesis. The MnFe_2_O_4_ nanocatalyst demonstrated impressive stability in recycling tests, maintaining its catalytic efficiency with only a slight decline after 5 uses.

## 3 Summary and outlook

Manganese-based magnetic catalysts have emerged as a highly efficient and sustainable class of catalysts, combining the redox versatility of manganese with the easy recoverability of magnetic materials. Their ability to participate in a wide range of catalytic processes—including oxidation reactions, organic transformations, electrocatalysis, and environmental applications—makes them a promising alternative to conventional catalysts. Compared to traditional homogeneous and heterogeneous catalysts, magnetic manganese catalysts offer a unique balance of high catalytic activity, stability, cost-effectiveness, and environmental sustainability.

### 3.1. Advantages of magnetic manganese catalysts

❖ Efficiency and versatility

✓ Manganese's multiple oxidation states (+2 to +7) enable diverse catalytic transformations, rivaling noble metal catalysts such as platinum and palladium.

✓ Functionalized Mn-based magnetic nanoparticles exhibit enhanced selectivity and activity in oxidation and cross-coupling reactions, making them competitive with traditional catalytic systems.

❖ Magnetic separation and reusability

✓ Unlike homogeneous catalysts, which are difficult to recover and often require extensive purification, magnetic manganese catalysts can be easily separated from reaction mixtures using an external magnetic field, reducing processing costs and minimizing catalyst loss.

✓ Their recyclability significantly enhances economic feasibility and long-term sustainability compared to non-magnetic heterogeneous catalysts.

❖ Environmental and economic benefits

✓ Manganese is earth-abundant and non-toxic, unlike precious metals such as ruthenium, iridium, and rhodium, which pose environmental and economic constraints.

✓ The use of Mn-based magnetic catalysts in green chemistry reduces reliance on toxic reagents and harsh reaction conditions, contributing to cleaner and more sustainable industrial processes.

❖ Stability and industrial applicability

✓ Magnetic manganese catalysts demonstrate high thermal and chemical stability, allowing them to be employed in a range of reaction conditions without significant deactivation.

✓ Their adaptability in water treatment, electrocatalysis, and pharmaceutical synthesis positions them as viable candidates for large-scale applications.

### 3.2. Comparative challenges and future perspectives

While magnetic manganese catalysts exhibit significant advantages, some challenges remain:

❖ Tuning catalyst selectivity: further optimization of Mn-functionalized surfaces is required to enhance selectivity for specific chemical transformations.

❖ Long-term stability: some Mn-based catalysts may suffer from leaching or deactivation over multiple cycles, necessitating improved material design.

❖ Scalability: although promising, large-scale production and integration into industrial processes require further research to optimize cost-effectiveness.

Future advancements in nanotechnology, surface engineering, and computational catalyst design will likely enhance the efficiency and applicability of magnetic manganese catalysts. Additionally, integrating these catalysts with renewable energy sources and sustainable reaction pathways could further cement their role in next-generation catalysis.

## 4 Final outlook

Magnetic manganese catalysts represent a transformative approach to modern catalysis, offering a unique synergy of catalytic efficiency, recoverability, and sustainability. When compared to traditional transition metal catalysts, they provide superior recyclability, cost advantages, and environmental benefits while maintaining high performance. As research in materials science and catalysis continues to evolve, magnetic manganese catalysts are expected to play a pivotal role in green chemistry, industrial catalysis, and energy conversion technologies, paving the way for more efficient and sustainable chemical processes.

## Conflicts of interest

The authors declare no conflict of interest.

## Data Availability

No primary research results, software or code have been included and no new data were generated or analyzed as part of this review.
